# Reduced Protein Import via TIM23 SORT Drives Disease Pathology in TIMM50-Associated Mitochondrial Disease

**DOI:** 10.1080/10985549.2024.2353652

**Published:** 2024-06-03

**Authors:** Jordan J. Crameri, Catherine S. Palmer, Tegan Stait, Thomas D. Jackson, Matthew Lynch, Adriane Sinclair, Leah E. Frajman, Alison G. Compton, David Coman, David R. Thorburn, Ann E. Frazier, Diana Stojanovski

**Affiliations:** a Department of Biochemistry and Pharmacology, The University of Melbourne, Parkville, Victoria, Australia; b The Bio21 Molecular Science and Biotechnology Institute, The University of Melbourne, Parkville, Victoria, Australia; c Murdoch Children’s Research Institute, Royal Children’s Hospital, Parkville, Victoria, Australia; d Victorian Clinical Genetics Services, Royal Children’s Hospital, Parkville, Victoria, Australia; e Neurosciences Department, Queensland Children’s Hospital, South Brisbane, Queensland, Australia; f Department of Metabolic Medicine, Queensland Children’s Hospital, South Brisbane, Queensland, Australia; g School of Medicine, University of Queensland, St Lucia, Queensland, Australia; h Department of Paediatrics, The University of Melbourne, Parkville, Victoria, Australia

**Keywords:** Mitochondria, mitochondrial disease, mitochondrial protein import, TIM23 complex, TIMM50

## Abstract

TIMM50 is a core subunit of the TIM23 complex, the mitochondrial inner membrane translocase responsible for the import of pre-sequence-containing precursors into the mitochondrial matrix and inner membrane. Here we describe a mitochondrial disease patient who is homozygous for a novel variant in *TIMM50* and establish the first proteomic map of mitochondrial disease associated with TIMM50 dysfunction. We demonstrate that TIMM50 pathogenic variants reduce the levels and activity of endogenous TIM23 complex, which significantly impacts the mitochondrial proteome, resulting in a combined oxidative phosphorylation (OXPHOS) defect and changes to mitochondrial ultrastructure. Using proteomic data sets from TIMM50 patient fibroblasts and a TIMM50 HEK293 cell model of disease, we reveal that laterally released substrates imported via the TIM23^SORT^ complex pathway are most sensitive to loss of TIMM50. Proteins involved in OXPHOS and mitochondrial ultrastructure are enriched in the TIM23^SORT^ substrate pool, providing a biochemical mechanism for the specific defects in TIMM50-associated mitochondrial disease patients. These results highlight the power of using proteomics to elucidate molecular mechanisms of disease and uncovering novel features of fundamental biology, with the implication that human TIMM50 may have a more pronounced role in lateral insertion than previously understood.

## Introduction

**Figure 1.**
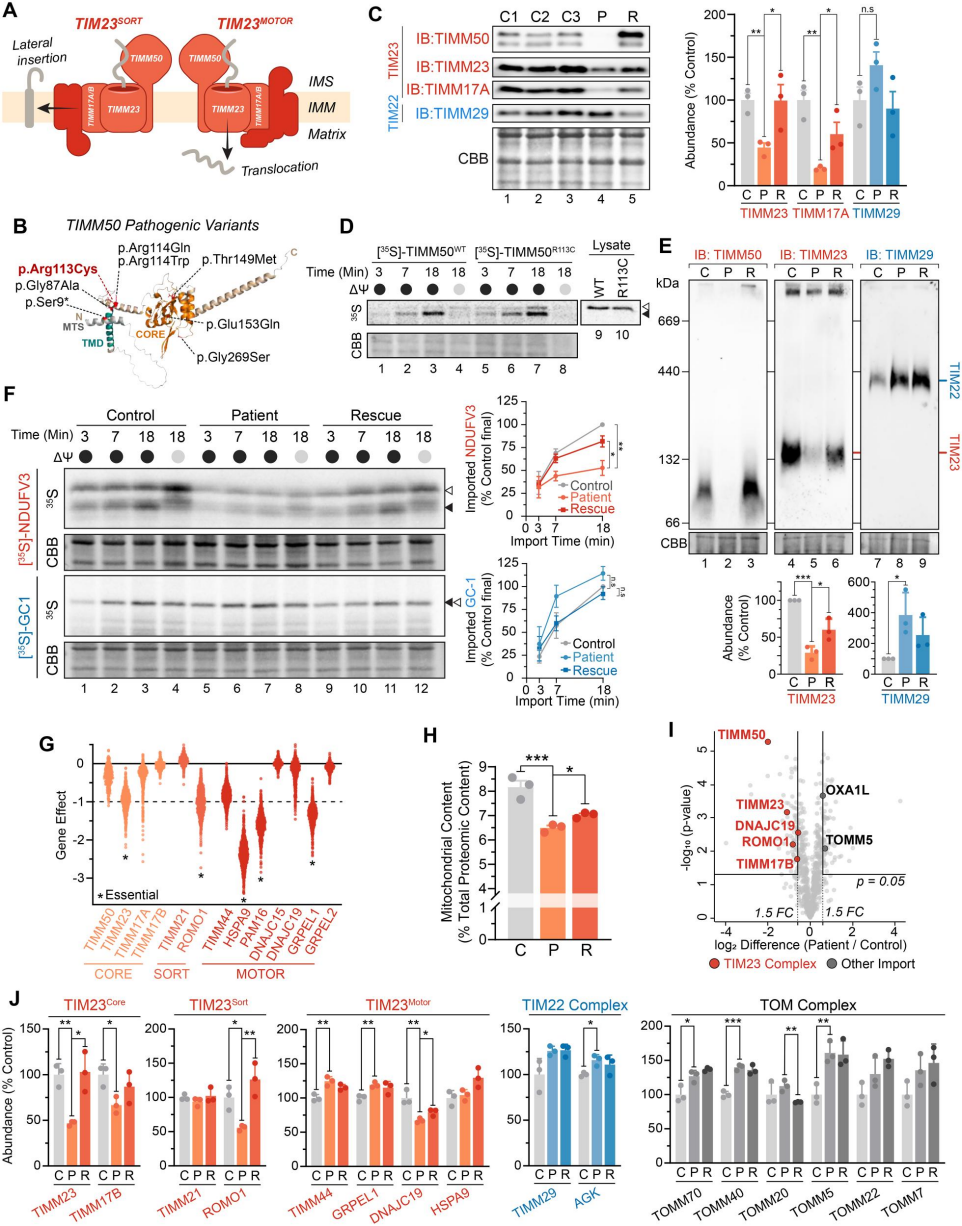
Isolated TIM23 defect in TIMM50 patient fibroblasts. (A) Depiction of the human TIM23 complex located in the inner mitochondrial membrane. TIM23 consists of core subunits TIMM23, TIMM50 and TIMM17A/B. TIM23^CORE^ associates with accessory subunits forming either TIM23^SORT^ for the lateral insertion of single or dual pass proteins into the inner membrane, or TIM23^MOTOR^ for the complete translocation of proteins into the mitochondrial matrix. IMS: intermembrane space, IMM: inner mitochondrial membrane. (B) Predicted AlphaFold^66^^,^^67^ structure (AF-Q3ZCQ8) of human TIMM50 colored according to protein domains (mitochondrial targeting sequence (MTS), transmembrane domain (TMD), FCP1-like/core domain (CORE)) and annotated with location of reported pathogenic variants as detailed in Supplementary Table 1. Variant identified in this study is denoted in red. All variants have been mapped to TIMM50 NCBI refseq protein NP_001001563.2. (C) SDS-PAGE and western blot of mitochondria isolated from fibroblast cells probing for TIM translocase subunits, using Coomassie staining as a loading control. Densitometric quantification (right) is presented as percentage of control average, normalized to Coomassie stained loading control. (D) In vitro import of ^35^S radiolabeled TIMM50^WT^ and TIMM50^R113C^ into mitochondria isolated from WT HEK293 cells in the presence (black circle) or absence (grey circle) of membrane potential (ΔΨ) dissipated with 10 μM FCCP. Following incubation for indicated times, all samples were treated with proteinase K. Coomassie staining is presented as loading control. Empty arrowhead = precursor, filled arrowhead = mature. (E) BN-PAGE and Western blot analysis of mitochondria isolated from fibroblast cells and solubilized in 1% digitonin probing for TIM translocases. Coomassie staining is presented as loading control. Densitometric quantification (below) is presented as percentage of control values, normalized to Coomassie stained loading control. (F) In vitro import of TIM23 substrate (NDUFV3) and TIM22 substrate (GC-1) into mitochondria isolated from fibroblast cells in the presence (black circle) or absence (gray circle) of membrane potential (ΔΨ) dissipated with 10 μM FCCP. Following incubation for indicated times, all samples were treated with proteinase K. Coomassie staining is presented as loading control. Empty arrowhead = precursor, filled arrowhead = mature. Quantifications (right) calculated as percentage of final control timepoint abundance normalized to Coomassie staining. (G) Gene effect scores of TIM23 subunit CRISPR knockouts in cancer cell lines (DepMap Public 23Q2 + Score, Chronos). Lower gene effect scores indicate increased likelihood of dependence in a cell line. A gene effect of −1 is the median score of all common essential genes. Essential gene status was manually curated from the DepMap portal. (H) Determination of mitochondrial protein content in whole cell proteomic data. Total label-free quantification (LFQ) values of mitochondrial proteins were summed and presented as a percentage of total LFQ values of all detected proteins within samples. (I) Quantitative proteomic volcano plot of isolated mitochondria from TIMM50 patient fibroblasts relative to controls. Labelled are significantly altered proteins involved in mitochondrial protein import pathways. (J) Translocase subunit abundance of detected proteins determined from isolated mitochondrial proteomics of fibroblast cells. Values are presented as a percentage of control averages. Data information: C, Control; P, TIMM50 Patient; R, TIMM50 Patient/TIMM50^WT^ Rescue; FC, fold-change; CBB, Coomassie brilliant blue; IB, immunoblot. In C, E, F, H and J, data are presented as mean ± SD. *P ≤ 0.05, **P < 0.01, ***P < 0.001, ****P < 0.0001, n.s = *P* > 0.05. *N* = 3 (unpaired Student’s *t* test).

Of the approximate 1,100 mitochondrial proteins in human cells, only 13 are encoded by the mitochondrial genome (mtDNA).^1^^,^^2^ The remainder are encoded by the nuclear genome and are imported into mitochondria by translocases embedded within the mitochondrial membranes.^3^ The translocases of the outer membrane (TOM complex) and inner membrane (TIM23 complex) coordinate to deliver approximately 60% of the mitochondrial proteome to the mitochondrial matrix and inner membrane.^4^^,^^5^ The TIM23 complex is a multifunctional translocase built around a core (comprised of TIMM23, TIMM50 and TIMM17A/B) that engages with discrete subsets of accessory subunits forming either TIM23^MOTOR^ or TIM23^SORT^ complexes (Figure 1A).^6^ TIM23^MOTOR^, formed by association with the motor-specific subunits DNAJC19/15, TIMM44, HSPA9, PAM16, GRPEL1 and GRPEL2, is responsible for the translocation of proteins into the mitochondrial matrix, whilst TIM23^SORT^, formed by association with the sort-specific subunits TIMM21 and ROMO1, facilitates the lateral insertion of single or dual pass membrane proteins into the inner membrane.^6^ The inner membrane also contains an additional translocase, the TIM22 complex, which is involved in the import of multispanning membrane proteins, including the abundant SLC25A carrier protein family into the inner membrane.^7^

TIM23 complex dysregulation is increasingly associated with mitochondrial diseases, a heterogeneous group of genetic disorders involving mitochondrial dysfunction.^7^^,^^8^ Pathological variants of the core TIM23 complex subunits *TIMM23 and TIMM17A/B* have not yet been described, but several reported cases of pathogenic variants in *TIMM50* have been connected to the mitochondrial disease 3-methylglutaconic aciduria, type IX (MGCA9; MIM: 617698) (Supplementary Table S1).^9–14^ MGCA9 is an early onset autosomal recessive disorder that is characterized by seizures, intellectual disability, and developmental delay (Supplementary Table S1). MGCA9 patients present with a combined deficiency of the mitochondrial oxidative phosphorylation (OXPHOS) complexes (I-V) that are responsible for aerobic energy production.^15^ Whilst clinical presentations vary depending on both tissue type and analysis method, pathogenic variants in *TIMM50* have been reported to impact all OXPHOS complexes except complex III.^9–11^

Based on the biochemical function of Tim50 in the yeast *Saccharomyces cerevisiae,* human TIMM50 is predicted to function as a receptor at the TIM23 complex, interacting with precursor proteins that contain mitochondrial targeting sequences (MTS) and guiding them to the pore of the translocase.^16–18^ Human TIMM50, however, has diverged from its yeast homolog, no longer possessing a key protein domain involved in precursor handover.^19^ Therefore, studies on human TIMM50 are required to clarify its function in higher organisms and elucidate the molecular mechanisms underlying mitochondrial dysfunction due to *TIMM50* pathogenic variants. Indeed, the role of TIMM50 in mitochondrial protein import evokes the question of whether TIMM50 dysfunction leads to a broad mitochondrial import defect via the TIM23 complex, or if there is a more localized impact on a subset of TIM23 complex substrates. The specific defect observed in OXPHOS complexes suggest the latter,^9^^,^^11^ but the mechanism(s) supporting this phenotype are unknown.

Here, we describe a mitochondrial disease patient homozygous for a novel variant in *TIMM50* with clinical symptoms consistent with previously reported cases of MGCA9. Assessment of patient fibroblasts demonstrated reduced levels and activity of the TIM23 complex, which impacts the mitochondrial proteome, resulting in a combined OXPHOS defect and changes to mitochondrial ultrastructure. Using quantitative proteomics of patient fibroblasts and a CRISPR-Cas9 mediated TIMM50 HEK293 cell model of disease, we have established the first proteomic map of mitochondrial disease associated with *TIMM50* pathogenic variants. Interrogation of this data set, specifically looking at the proteomic impact on TIM23^MOTOR^ versus TIM23^SORT^ substrates, in addition to protein half-lives in control versus patient cells, suggests that mitochondrial complexes with high turnover and dependence on TIM23^SORT^ are disproportionately affected by *TIMM50* pathogenic variants. This specifically impacts several mitochondrial complexes that function in energy production and mitochondrial ultrastructure, providing an explanation for the observed defects in patient cells. This work demonstrates the power of coupling patient cell biology with proteomics to help elucidate molecular mechanisms of disease and uncover novel features of fundamental biology.

## Results

### Identification of a novel pathogenic variant in TIMM50

A homozygous missense variant in exon 5 of *TIMM50* (NM_001001563.5: c.337C > T) was identified by rapid exome sequencing in a patient born to consanguineous parents who presented with global developmental delay, optic nerve atrophy, generalized white matter loss and infantile spasms. While these features are consistent with other reported *TIMM50* patients, urinary excretion of 3-methylglutaconic acid (3-MGA) was not detected in this patient. A summary of the clinical characteristics of the patient described in this study and other published *TIMM50* patients can be found in Supplementary Table S1. A detailed clinical case report can also be found in the supplementary material.

The c.337C > T *TIMM50* variant was predicted to result in an amino acid change from an arginine to a cysteine at amino acid position 113 on the intermembrane space facing side of the protein after the single-pass transmembrane domain (NP_001001563.2; p.(Arg113Cys)) (Figure 1B). The arginine residue at this position has high conservation (100 vertebrates track, UCSC genome browser) and in silico predictions for the variant were consistently pathogenic (PolyPhen, SIFT, CADD, MutationTaster) and rare, with a minor allele frequency of 0.00001053 in the gnomAD v4.0 database (0 homozygotes). Whilst this variant is novel, variants in the adjacent arginine residue (p.(Arg114Trp) and p.(Arg114Gln)), have been reported in patients with MGCA9 (Figure 1B) (Supplementary Table S1). Of note, many of the previously reported *TIMM50* variants were mapped to a longer *TIMM50* isoform which was predicted to have a nuclear localization signal.^20^ The canonical RefSeq transcript has since been updated to the shorter mitochondrial isoform, and all reported variants were remapped accordingly (Supplementary Table S1). In line with previous investigations of TIMM50 patient fibroblasts,^9^ this study focused solely on the canonical shorter mitochondrial TIMM50 isoform. Based on the information available at the time of identification, the variant reported here was classified as a variant of uncertain significance with potential clinical relevance. Therefore, additional biochemical analysis was conducted to provide support for its pathogenicity.

### TIM23 complex function is impaired in TIMM50 patient fibroblasts

The homozygous p.(Arg113Cys) variant in TIMM50 resulted in near-complete protein loss in mitochondria isolated from patient fibroblasts on SDS-PAGE (Figure 1C). This coincided with significant reductions in the steady state levels of the core TIM23 complex subunits TIMM23 and TIMM17A (Figure 1C). Prolonged stable re-expression of TIMM50^WT^ for 5 days resulted in improved abundances of both TIMM23 and TIMM17A (Figure 1C and Supplementary Figure S1A). The TIM22 complex subunit TIMM29, which functions independently of the TIM23 complex, was unaffected (Figure 1C). In vitro import of TIMM50^WT^ and the TIMM50 patient variant (TIMM50^R113C^) into mitochondria isolated from WT HEK293 cells demonstrated comparable import kinetics and precursor processing, suggesting TIMM50^R113C^ is capable of import into the organelle and the observed protein loss is not due to lack of import (Figure 1D). Blue native (BN)-PAGE analysis of mitochondrial protein complexes demonstrated a significant reduction in assembled TIM23 complex abundance (Figure 1E). Re-expression of TIMM50^WT^ significantly increased TIM23 complex abundance, whilst elevated TIM22 complex levels in the patient were unchanged (Figure 1E). To determine if the observed reduction in TIM23 complex impacted mitochondrial protein import rates, in vitro import assays were conducted with radiolabeled TIM23 ([^35^S]-NDUFV3) and TIM22 complex ([^35^S]-GC1) substrates. Mitochondria isolated from TIMM50 patient fibroblasts had reduced import kinetics for [^35^S]-NDUFV3 that improved to near control levels with TIMM50^WT^ re-expression (Figure 1F). The import of the TIM22 complex substrate [^35^S]-GC1 was unaffected in mitochondria isolated from TIMM50 patient cells (Figure 1F). The persistence of import via the TIM22 complex indicated that the patient fibroblasts retained sufficient membrane potential to facilitate protein import in vitro.

A significant proportion of the mitochondrial proteome is imported via the TIM23 complex, with numerous TIM23 complex subunits classified as being essential for cell viability (Figure 1G). The cellular context of the TIMM50 patient fibroblasts, whereby TIM23 complex levels are significantly reduced but cell viability is maintained, provided a unique opportunity to investigate the consequences of TIM23 complex dysregulation on broader mitochondrial function in human cells. To facilitate this investigation, we employed a proteomic pipeline for the unbiased assessment of TIMM50 patient fibroblasts in both whole cell and isolated mitochondrial samples (Supplementary Figure S1B). Whole cell proteomics revealed that the total cellular mitochondrial protein content was reduced from approximately 8% to 6.5% in patient cells relative to controls, consistent with impaired mitochondrial protein import in TIMM50 patient fibroblasts (Figure 1H). Re-introduction of TIMM50^WT^ only partially increased mitochondrial content, perhaps due to insufficient time of rescue. Mitochondrial proteomics uncovered broad alterations to the mitochondrial proteome, with proteins involved in key mitochondrial processes including OXPHOS and cristae architecture reduced relative to control fibroblasts (Supplementary Figure S1C and D). Whole cell proteomics reaffirmed reductions in OXPHOS and cristae related proteins, in addition to the significant upregulation of nonmitochondrial proteins involved in DNA, RNA and protein synthesis (Supplementary Figure S1E and F). Mitochondrial proteomics revealed reductions in the steady-state levels of core TIM23 complex subunits, with the TIM23^SORT^ subunit ROMO1 and the TIM23^MOTOR^ subunit DNAJC19 being the only noncore subunits significantly reduced (Figure 1I and J). Components of other mitochondrial import translocases (TIM22 and TOM) were either unaffected or slightly elevated (Figure 1J). These data suggest that defects in TIMM50 patient cells are driven by reduced TIM23 complex levels and consequently TIM23 mediated protein import, resulting in broad impacts on the mitochondrial proteome, with a particular impact on OXPHOS and mitochondrial organization.

### Combined OXPHOS deficiency upon loss of TIMM50 function

**Figure 2.**
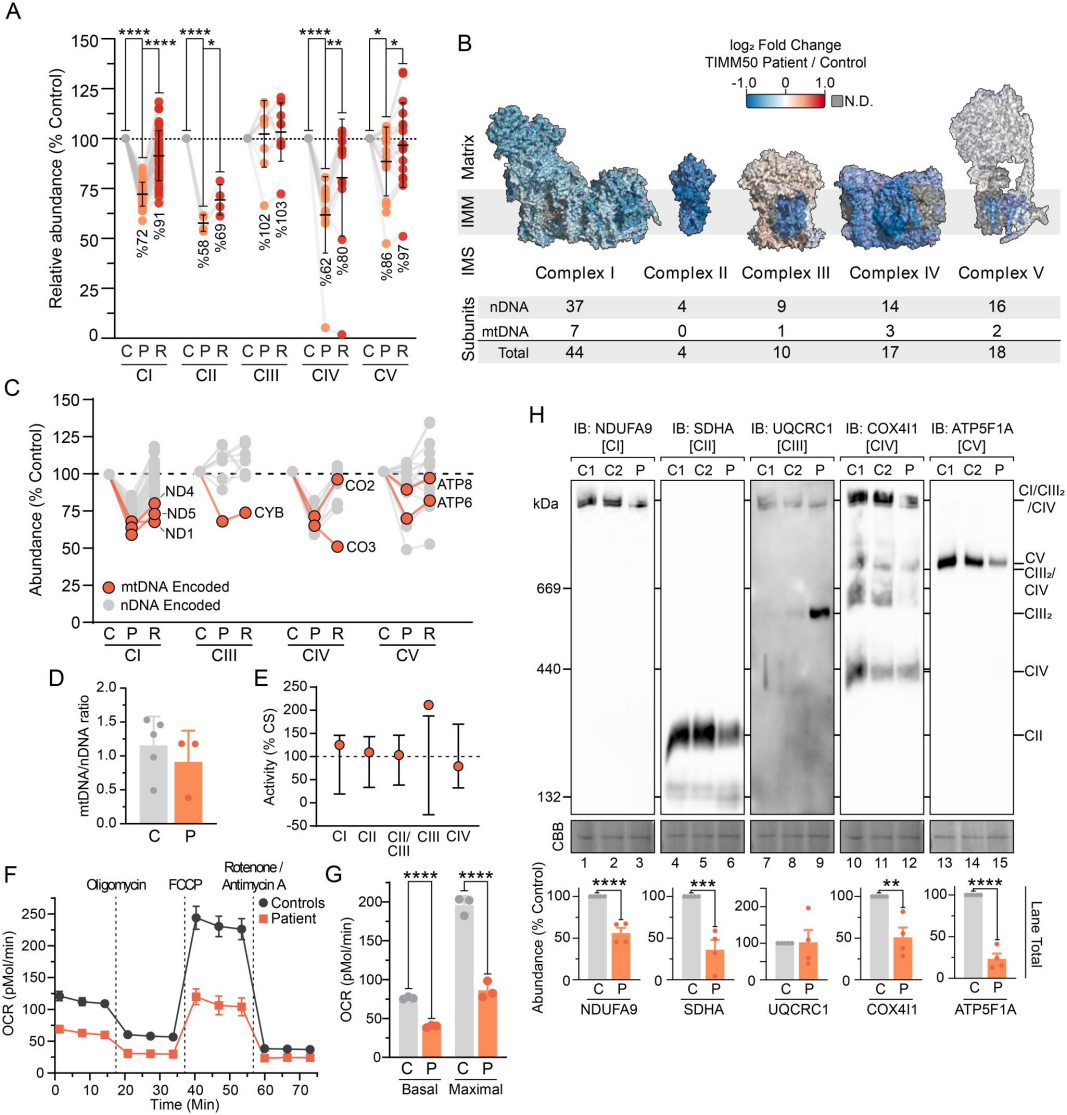
Combined OXPHOS deficiency in TIMM50 patient fibroblasts. (A) Relative abundance of OXPHOS complexes in fibroblast cells as determined from isolated mitochondria proteomics. Each data point represents an individual OXPHOS complex subunit. Complex abundance is calculated as the average abundance of all complex subunits. Percentage values represent complex averages. (B) Mapping of OXPHOS complex subunit fold changes (as shown in A) of TIMM50 patient fibroblasts relative to controls onto complex structures for visualization. Included below are details of complex subunit genomic source. N.D. = not detected. (C) Abundance of mtDNA encoded OXPHOS complex subunits in TIMM50 patient fibroblasts relative to controls as determined from isolated mitochondria proteomics. Each data point represents an individual OXPHOS complex subunit. Gray dots indicate subunits encoded by nuclear DNA. (D) Quantitative PCR determination of mtDNA relative to nuclear DNA (nDNA) in TIMM50 patient and control fibroblast cell lines. *N* = 3 subcultures (TIMM50 patient cell line) or *N* = 5 control cell lines. (E) Individual OXPHOS complex enzymology in TIMM50 patient fibroblasts as a percentage of citrate synthase (CS) activity. Error bars indicate range of control fibroblast values. (F and G) Oxygen consumption rate (OCR) measured in live fibroblast cells using a Seahorse XFe96 Analyzer following injection of indicated inhibitors. Control data are presented as average of 5 individual controls. Presented in G are quantifications of basal (prior to oligomycin treatment) and maximal mitochondrial OCR (following FCCP treatment). (H) BN-PAGE of mitochondria isolated from control and TIMM50 patient fibroblast cells solubilized in 1% digitonin and probed for OXPHOS complexes as indicated. Coomassie staining is presented as loading control. Densitometric quantification for total lane intensity (below) is presented as percentage of control values, normalized to Coomassie staining loading control. Data information: C, Control; P, TIMM50 Patient; R, TIMM50 Patient/TIMM50^WT^ Rescue; CBB, Coomassie brilliant blue; IB, immunoblot. In A, D, F, G, and H, data are presented as mean ± SD. *P ≤ 0.05, **P < 0.01, ***P < 0.001, ****P < 0.0001, *N*  = 3 (For A: paired Student’s *t* test, For G and H: unpaired Student’s *t* test). In (E), error bars indicate range of control values.

The TIM23 complex subunit ROMO1 regulates the distribution of TIMM21 between TIM23^SORT^ complexes and MITRAC (mitochondrial translation regulation assembly intermediate of cytochrome *c* oxidase) complexes during the assembly of complex IV.^21^ Our proteomic identification of reduced ROMO1 abundance (Figure 1I and J) in conjunction with the downward trend in OXPHOS proteins (Supplementary Figure S1D and F) in the TIMM50 patient fibroblasts prompted a more targeted OXPHOS complex assessment. Consistent with the literature,^9^^,^^11^ TIMM50 patient fibroblasts had significant reductions in the abundances of complexes I, II and IV, whilst complex V subunits had variable abundances and complex III was unaffected (Figure 2A and B and Supplementary Figure S2A). The abundance of complexes I, II and IV increased with re-expression of TIMM50^WT^, with the improvement in complexes I and IV being greater than that of complex II (Figure 2A). Notably, across all OXPHOS complexes, every mtDNA encoded subunit was decreased in abundance despite the level of mtDNA in the patient fibroblasts being comparable to controls (Figure 2C and D). Whilst the reduced levels of mtDNA-encoded subunits undoubtedly contribute to the observed OXPHOS defects, it alone cannot adequately explain the specific combined OXPHOS deficiency. For example, complex II, which contains no mtDNA encoded subunits, was the most reduced OXPHOS complex, while complex III, which does contain an affected mtDNA encoded subunit, was otherwise unchanged in total complex abundance (Figure 2A to C). Likewise, every OXPHOS complex is also reliant on the import of cytosolically synthesized subunits, and there is no common cofactor or assembly pathway shared by complexes I, II and IV that could explain their specific reductions in abundance (Figure 2B). Despite these changes to steady state OXPHOS complex abundances, the enzymatic activities of the individual complexes were unaffected in enriched mitochondrial preparations from TIMM50 patient fibroblasts (Figure 2E and Supplementary Table S2). Discrepancies between OXPHOS complex abundance and activity are not uncommon and have been reported previously in TIMM50 patients.^9^^,^^11^ Likewise, pediatric mitochondrial disease patients that have OXPHOS enzymatic defects in skeletal muscle frequently have normal enzyme activities in fibroblasts.^22^ However, in line with reduced OXPHOS complex abundances, measurement of cellular respiration in TIMM50 patient fibroblasts demonstrated that both basal and maximal oxygen consumption rates were significantly reduced relative to control fibroblasts (Figure 2F and G).

BN-PAGE reaffirmed reduced abundance of complexes I, II and IV whilst complex V presented with a more severe defect than was apparent via mass spectrometry (Figure 2H). The use of the mild detergent digitonin with BN-PAGE preserves complexes I, III and IV in higher-order structures called supercomplexes.^23^^,^^24^ Accordingly, whilst the total abundance of complex III was unchanged in TIMM50 patient fibroblasts, the reduced level of complexes I and IV lead to complex III being stabilized in the independent dimeric form (Figure 2H). Re-expression of TIMM50^WT^ reduced the level of dimeric complex III implying a direct connection with TIMM50 abundance (Supplementary Figure S2B). It is unclear if this stabilized complex III represents fully assembled complex III dimer, especially due to reduced abundance of late-stage complex III assembly factors BCS1L and TTC19 (Supplementary Figure S2C). The persistence of complex III dimers upon supercomplex disruption has been previously documented, and complexes I and IV are known to have high interdependency for stability.^25^ Therefore, it is difficult to determine if the observed defects in complexes I and IV are both primary defects or if one complex is destabilized upon the loss of the other. Whilst we did not observe a significant defect in MITRAC assembly factors (Supplementary Figure S2D), the significant loss of ROMO1 may be interfering with TIMM21 partitioning between TIM23 complex and MITRAC complexes during complex IV biogenesis.^21^ The mechanism underlying the loss of complex II in TIMM50 patient fibroblasts in this study and other published work is unclear.^9^^,^^11^ Despite no known biochemical connections, loss of ROMO1 has been shown to reduce complex II steady state abundance and activity.^21^ The membrane embedded complex II subunits SDHC and SDHD are predicted to be imported via a TIM23 complex pathway involving both TIM23^SORT^, TIM23^MOTOR^, and OXA1L machinery.^26^^,^^27^ Thus, the observed defect in complexes I, II and IV independent of complexes III and V may be driven by the loss of ROMO1 and associated import pathways in TIMM50 patient fibroblasts.

### TIMM50 pathogenic variants compromise inner membrane integrity

**Figure 3.**
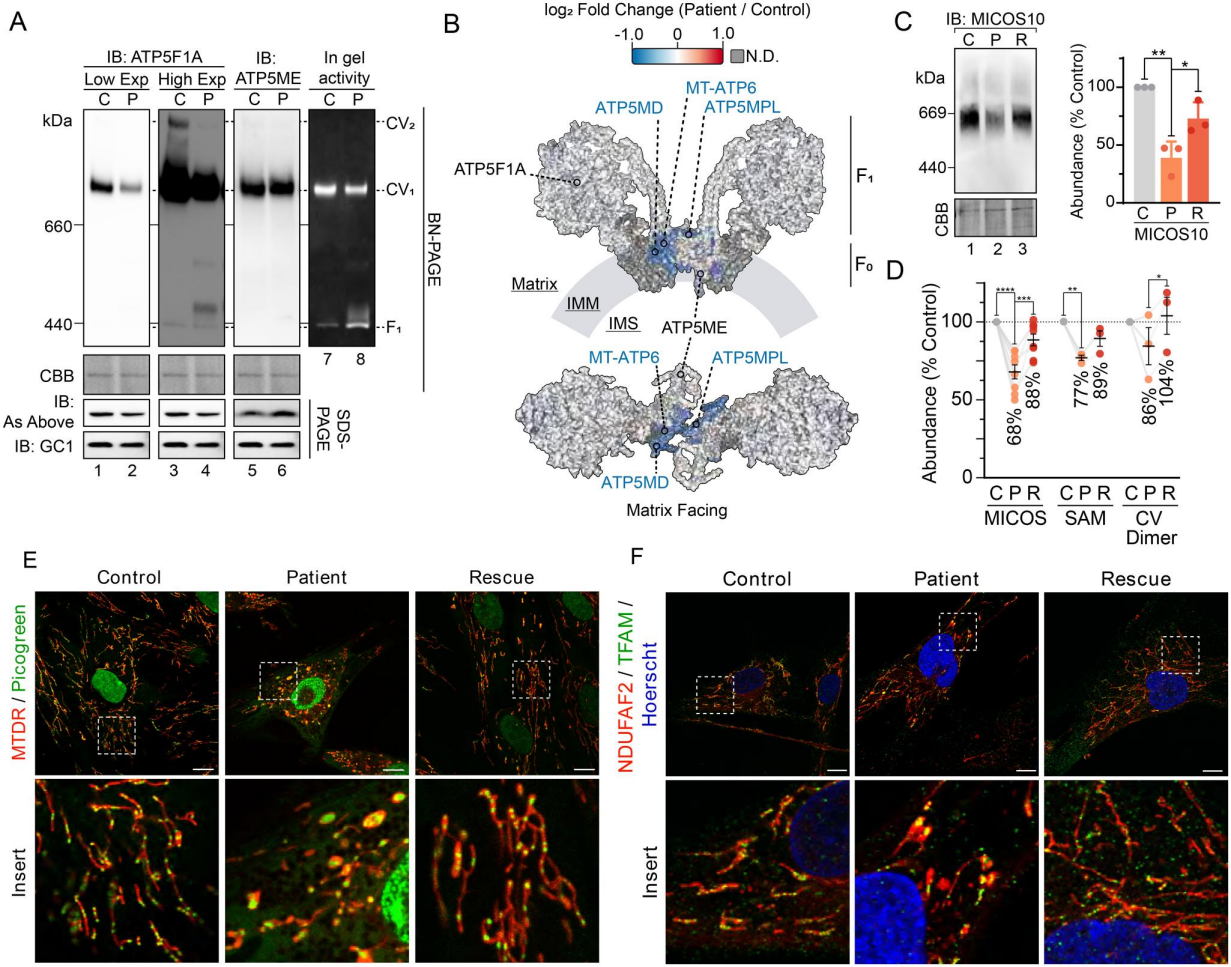
Compromised inner membrane integrity with pathogenic variants in TIMM50. (A) Immunoblotting and in-gel activity of complex V assessed via BN-PAGE and SDS-PAGE of mitochondria isolated from control and TIMM50 patient fibroblast cells. BN-PAGE samples were solubilized in 1% digitonin. “IB: As Above” indicates usage of the same antibody on BN-PAGE as on SDS-PAGE. Coomassie staining was used as a loading control for BN-PAGE. GC1 was used as a loading control for SDS-PAGE. (B) Mapping of complex V subunit fold changes (as shown in Figure 2A) onto the structure of dimerized complex V (PDB: 7AJD). Labelled are significantly affected subunits, relevant subunits for immunoblotting in Figure 3A, and subcomplex regions. N.D. = not detected. (C) BN-PAGE of mitochondria isolated from fibroblast cells and solubilized in 1% digitonin. Coomassie staining is presented as loading control. Densitometric quantification (right) is presented as percentage of control values, normalized to Coomassie staining loading control. (D) Relative abundance of mitochondrial complexes in fibroblast cells as determined from isolated mitochondria proteomics. Each data point represents an individual complex subunit. Complex abundance is calculated as the average abundance of all complex subunits. Percentage values represent complex averages. (E) Confocal microscopy of fibroblasts immunostained for mitochondria (NDUFAF2, mitochondrial matrix) and mitochondrial DNA (TFAM). Nucleus was stained with Hoechst. Scale bars = 10 μm, Inset shows enlargement of boxed area. (F) Immunofluorescence confocal microscopy of fibroblasts fixed with paraformaldehyde. Nuclei were stained with DAPI and mitochondria with mitochondrial matrix protein NDUFAF2. Immunostaining of TFAM was used for mtDNA visualization. Data information: C, Control; P, TIMM50 Patient; R, TIMM50 Patient/TIMM50^WT^ Rescue; CBB, Coomassie brilliant blue; Exp, exposure; IB, immunoblot. In C and D, data are presented as mean ± SD. *P ≤ 0.05, **P < 0.01, ***P < 0.001, ****P < 0.0001, *N* = 3 (For C: unpaired Student’s *t* test, For D: paired Student’s *t* test).

Differences in the abundance of complex V by BN-PAGE analysis and mass spectrometry prompted a closer examination of the observed defect, as variable deficiency in complex V has been reported in TIMM50 patients.^10^ Additional analysis of complex V via BN-PAGE revealed that the apparent complex V changes were specific to the subunit used for immunodetection (Figure 3A). Whereas a reduction in assembled complex V was observed when immunoblotting with antibodies targeting the F_1_ domain subunit ATP5F1A (Figures. 2H and 3A), assessment via the membrane-embedded F_0_ domain protein ATP5ME showed no change (Figure 3A). Crucially, the steady-state abundance of these two subunits by SDS-PAGE and proteomics were unchanged (Figure 3A and B). In-gel complex V activity was slightly reduced in patient fibroblasts and demonstrated increased free F_1_ domain activity (Figure 3A). Furthermore, complex V dimer was also absent when probed with ATP5F1A (Figure 3A). Mapping the mass spectrometry determined subunit fold changes onto the structure of the complex V dimer revealed that all significantly affected subunits were concentrated at the dimer interface (Figure 3B). Disrupted complex V dimerization may explain the variable complex V abundance observed by BN-PAGE, whereby certain complex V sub-complexes may be more sensitive to digitonin solubilization with the absence of dimerization. Indeed, the presentation of complex V defects on BN-PAGE is known to vary with different antibodies without observed changes to overall complex V migration.^28^^,^^29^ Alternatively, assembly factors involved in complex V F_1_ domain formation (ATPAF1 and ATPAF2) were significantly reduced in TIMM50 patient fibroblasts and could be contributing to the observed phenotype (Supplementary Figure S2C). Whilst F_1_ subunits were unchanged in abundance (Figure 3A and B), their incorporation into mature complex V may be reduced due to the decreased levels of these assembly factors. Ultimately, the manifestation of complex V defects in TIMM50 patient fibroblasts appears to be caused by complex instability/assembly defects, rather than defects in subunit abundances.

Complex V dimers are important for the curvature of the mitochondrial inner membrane and the generation and maintenance of cristae.^30^ Accordingly, we queried if there were changes to additional complexes involved in mitochondrial ultrastructure in the TIMM50 patient fibroblasts. Inner membrane cristae junctions are stabilized by the mitochondrial contact site and cristae organizing system (MICOS),^31^ which interacts with the outer membrane localized sorting and assembly machinery (SAM) to form the mitochondrial intermembrane space bridging (MIB) complex.^32^ Consistent with loss of complex V dimers, MICOS and SAM complex abundances were significantly reduced in the TIMM50 patient cells (Figure 3C and D). The loss of MIB complexes may disrupt the formation of cristae, subsequently impacting the dimerization of complex V. Alternatively, disruption of complex V dimers may impact membrane curvature and thus, the stability of MIB complexes. Whilst it is difficult to discern the primary effect, given the changes to complex V and MIB complexes, we subsequently assessed mitochondrial morphology in the TIMM50 patient cells by live-cell imaging using MitoTracker deep red (MTDR) to stain mitochondria and PicoGreen to stain mtDNA (in addition to nuclear DNA). This revealed the presence of large, swollen mitochondria in the TIMM50 patient cells and the accumulation of mtDNA in defined clusters (Figure 3E). Immunostaining of mtDNA in fixed cells using antibodies against the mtDNA associated protein TFAM demonstrated the same phenotype (Figure 3F). In healthy mitochondria, mtDNA nucleoids are distributed throughout the mitochondrial network to ensure even translation of mtDNA transcripts and partitioning of mtDNA during fission and fusion events.^33^ Cristae play a vital role in the distribution of mtDNA nucleoids, with loss of cristae causing mitochondrial swelling and mislocalization of mtDNA nucleoids.^34–36^ The combined observations of reduced complex V dimerization, reduced MIB complex assembly, and the appearance of swollen mitochondria with mtDNA accumulation are suggestive of a cristae defect in the TIMM50 patient fibroblasts. Whether this defect represents a secondary effect driven by defective protein import or involves a more direct role for TIMM50 in cristae formation is unclear.

### HEK293 cell models of disease recapitulate TIMM50 patient fibroblast phenotypes

**Figure 4.**
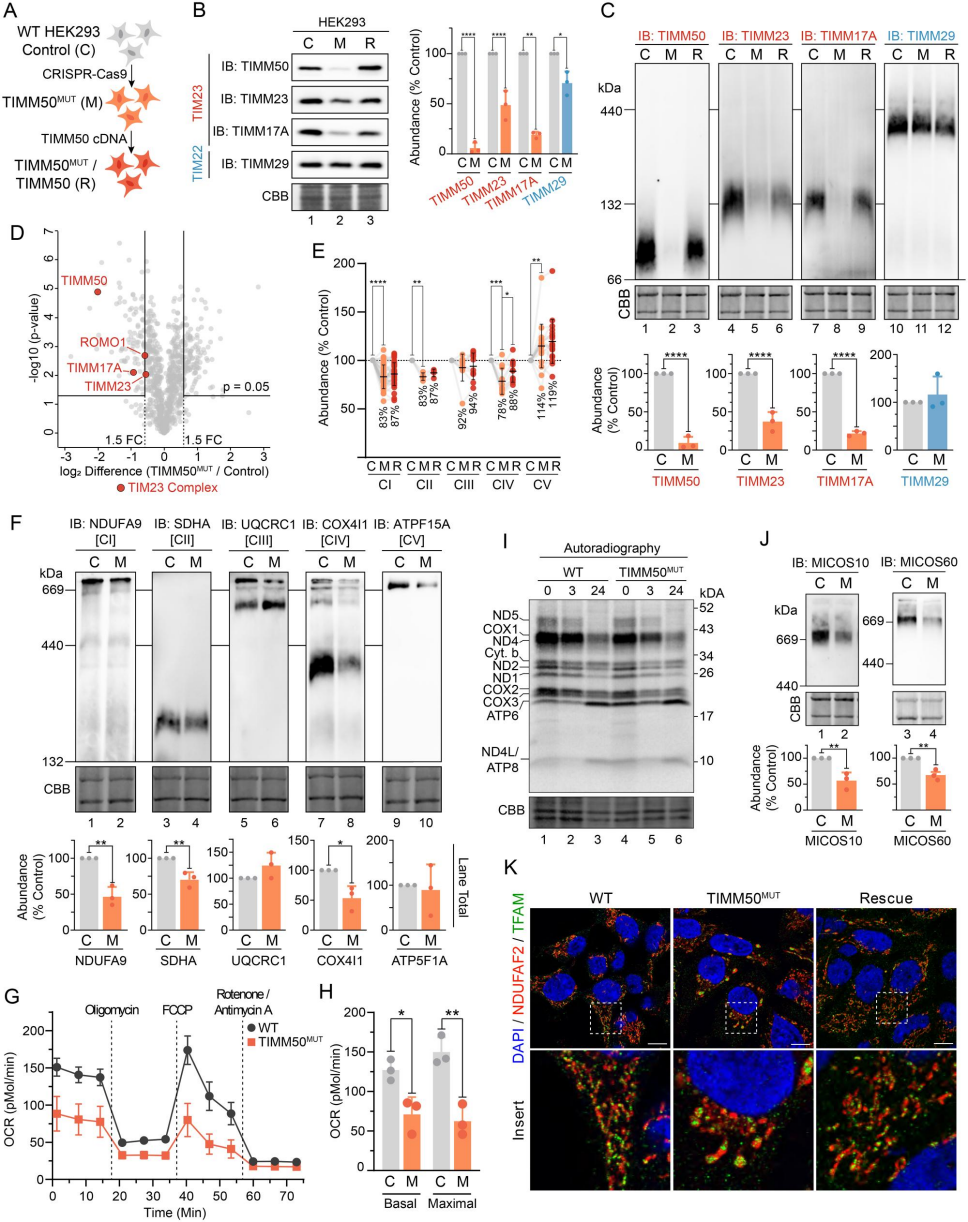
HEK293 models recapitulate mitochondrial defects. (A) Cell line generation and identities for HEK293 cell models of TIMM50 dysfunction. (B) SDS-PAGE of mitochondria isolated from HEK293 cells subjected to CRISPR-Cas9 gene disruption of TIMM50 and re-expression of WT TIMM50, probing for TIM translocase subunits. Coomassie staining is presented as loading control. Densitometric quantification (right) is presented as percentage of control average, normalized to Coomassie staining loading control. (C) BN-PAGE of mitochondria isolated from HEK293 cells solubilized in 1% digitonin probing for TIM translocase. Coomassie staining is presented as loading control. Densitometric quantification (below) is presented as percentage of control values, normalized to Coomassie staining loading control. (D) Quantitative proteomics volcano plot of isolated mitochondria from HEK293 TIMM50^MUT^ cells relative to WT controls. Labelled are significantly altered proteins involved in mitochondrial protein import. (E) Relative abundance of OXPHOS complexes in HEK293 cells as determined from whole cell proteomics. Each data point represents an individual OXPHOS complex subunit. Complex abundance is calculated as the average abundance of all complex subunits. Percentage values represent complex averages compared to controls. (F) BN-PAGE of mitochondria isolated from HEK293 cells solubilized in 1% digitonin probing for OXPHOS complexes. Coomassie staining is presented as loading control. Densitometric quantification (below) is presented as percentage of control values, normalized to Coomassie staining loading control. (G and H) Oxygen consumption rate (OCR) measured in live HEK293 cells using a Seahorse XFe96 Analyzer following injection of indicated inhibitors. Presented in H are quantifications of basal (prior to oligomycin treatment) and maximal mitochondrial OCR (following FCCP treatment). (I) Radiolabeling of mitochondrial translation products in HEK293 cells performed by pulsing with [^35^S]-Met/Cys for 2 h in the presence of the cytosolic translation inhibitor anisomycin followed by a chase for the indicated times. Isolated mitochondria were analyzed by SDS-PAGE and autoradiography. Coomassie staining is presented as a loading control. (J) BN-PAGE of mitochondria isolated from HEK293 cells and solubilized in 1% digitonin. Coomassie staining is presented as loading control. Densitometric quantification (below) is presented as percentage of control values, normalized to Coomassie staining loading control. (K) Confocal microscopy of HEK293 immunostained for mitochondria (NDUFAF2, mitochondrial matrix) and mitochondrial DNA (TFAM). Nuclei were stained with Hoechst. Inset shows enlargement of boxed area, scale bar = 10 μm. Data information: C, WT HEK293 Control; M, HEK TIMM50^MUT^; R, TIMM50^MUT^/TIMM50 rescue; CBB, Coomassie brilliant blue; FC, fold change; IB, immunoblot. In B, C, E–H, and J, data are presented as mean ± SD. *P ≤ 0.05, **P < 0.01, ***P < 0.001, ****P < 0.0001, *N* = 3 (For B, C, D, H and J: unpaired Student’s *t* test, For E: paired Student’s *t* test).

We generated a CRISPR-Cas9 *TIMM50* knockout (KO) cell line in Flp-In™ T-Rex™ 293 cells (HEK293), chosen for their high expression of TIMM50 and TIM23 complex subunits (Supplementary Figure S3A), to validate key observations made with the TIMM50 patient fibroblasts. Despite several attempts, we were unable to generate a complete KO of *TIMM50,* but were able to generate a cell line, designated TIMM50^MUT^ (MUT = mutant (M); Figure 4A and Supplementary Figure S3B) with significantly reduced TIMM50 level, confirmed by SDS-PAGE, BN-PAGE, mass spectrometry and immunofluorescence (Figure 4B to D and Supplementary Figure S3C). As observed in the TIMM50 patient fibroblasts, TIMM50^MUT^ cells had significantly reduced core TIM23 complex subunit abundances (Figure 4B and D, and Supplementary Figure S4A). BN-PAGE further demonstrated reduced abundance of assembled TIM23 complex whilst TIM22 complex levels were unchanged (Figure 4C). Significant reduction in the levels of ROMO1 via mitochondrial proteomics was apparent (Figure 4D), along with slight reductions in the levels of DNAJC19 and elevated TIMM44 levels detected via whole cell proteomics (Supplementary Figure S4A). Re-expression of TIMM50^WT^ rescued the levels of core TIM23 complex subunits and elevated the levels of TIM23^SORT^ (TIMM21 and ROMO1) and TIM23^MOTOR^ (TIMM44 and DNAJC19) subunits (Supplementary Figure S4A). Proteomic analysis confirmed that TIM22 and TOM complex subunits remained unchanged in TIMM50^MUT^, confirming a specific defect in TIM23 complex abundance (Supplementary Figure S4B and C). Total mitochondrial protein content was slightly reduced, but not to the extent seen in the TIMM50 patient fibroblasts (Supplementary Figure S4D vs Figure S1H).

As observed in the TIMM50 patient cells, TIMM50^MUT^ cells presented with a combined OXPHOS defect in complexes I, II and IV via BN-PAGE and mass spectrometry (Figure 4E and F). Complex III levels were unchanged, and complex V appeared upregulated via mass spectrometry and had high variability on BN-PAGE (Figure 4E and F). Supercomplex formation was also reduced and coincided with increased appearance of free complex III dimers, as was observed in the TIMM50 patient fibroblasts (Figure 4F). The significant variation in complex V on BN-PAGE despite elevated steady state levels via mass spectrometry is further evidence that complex V defects are stability/solubilization related, and do not represent reduced abundance (Figure 4E and F). Both basal and maximal oxygen consumption rates were significantly reduced in the TIMM50^MUT^ cells (Figure 4G and H), consistent with the reduced abundances of multiple OXPHOS complexes. The amount of mtDNA in the TIMM50^MUT^ cells was unchanged (Supplementary Figure S4E) and the translation of mtDNA encoded OXPHOS subunits was equivalent to WT control cells (Figure 4I). These data suggest that defects in mtDNA are not responsible for the observed reduction in OXPHOS complex abundances and respiration in the TIMM50^MUT^ cells.

Like TIMM50 patient cells, reduced abundance of ROMO1 in the TIMM50^MUT^ HEK293 cells did not coincide with a defect in MITRAC assembly factors (Supplementary Figure S4F). However, unique to the HEK293 TIMM50^MUT^ cells, was the significant reduction in levels of numerous complex II assembly factors, including SDHAF2, SDHAF3 and SDHAF4 (Supplementary Figure S4G). These complex II assembly factors were not detectable by mass spectrometry in either control or TIMM50 patient fibroblasts (Supplementary Figure S2C). Therefore, while their decrease may contribute to the reduction in complex II observed in patient cells, we cannot make definitive conclusions. Nevertheless, in both fibroblasts and HEK293 cells, there were changes to the distribution and migration of complex II sub-complexes on BN-PAGE (Supplementary Figure S4H). These sub-complexes represent interactions between complex II subunits and assembly factors,^37^ and their alteration may implicate complex II assembly factors in the observed defect.

The level of MICOS complex in TIMM50^MUT^ cells was significantly reduced (Figure 4J) and immunostaining of mitochondria with the matrix protein NDUFAF2 revealed large, swollen mitochondria consistent with the patient fibroblast phenotype (Figure 4K). TFAM immunostaining of mtDNA demonstrated perturbed localization of mtDNA with accumulation in swollen regions (Figure 4K). As with the TIMM50 patient fibroblasts, this phenotype suggests that the TIMM50^MUT^ HEK293 cells harbor a cristae defect. Together, these data demonstrated that the phenotype of the HEK293 TIMM50^MUT^ cells mirrored that of the TIMM50 patient fibroblasts, supporting the phenotypic observations as being specific to TIMM50 dysfunction. Ultimately, it can be concluded that the loss of TIMM50 reduces TIM23 complex abundance, resulting in the manifestation of a combined OXPHOS deficiency and perturbations to mitochondrial cristae, due to impaired protein import via the TIM23 complex.

### TIM23^SORT^ import is more significantly impacted than TIM23^MOTOR^ import upon loss of TIMM50

**Figure 5.**
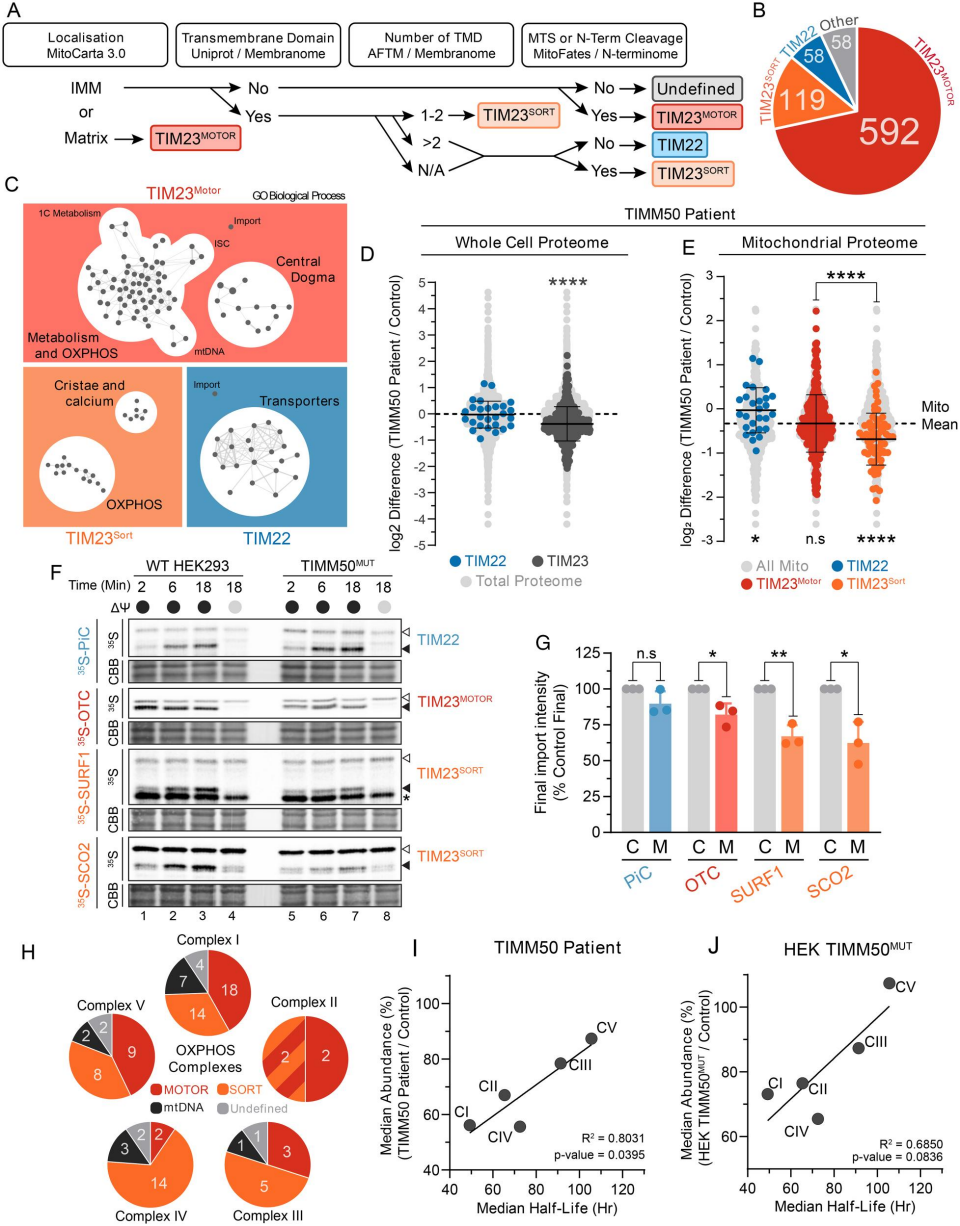
Mitochondrial pathways with increased TIM23^SORT^ dependence are most impacted by loss of TIMM50. (A) Pipeline for import pathway annotation of inner membrane and matrix proteins. Gene list of inner membrane and matrix proteins according to MitoCarta 3.0 (excluding mtDNA encoded proteins) were annotated with the following datasets; Uniprot, predicted transmembrane domain; Membranome, predicted transmembrane domain; AFTM, predicted number of transmembrane domains; Membranome, bitopic; MitoFates, mitochondrial targeting sequence prediction; N-terminome, presence of cleaved n-terminal sequence. Thresholds for each group were as follows; Transmembrane Domain, yes in either database; Number of transmembrane domains, prioritized Membranome bitopic; MTS or N-Term Cleavage, MitoFates score > 0.250 or presence of n-terminal cleavage. Following annotation, substrates predicted to be OXA1L substrates were manually re-assigned. (B) Breakdown of proteins assigned to each import pathway annotation. (C) Enrichment maps of GO biological process terms from import pathway annotation data sets with false discovery rate of less than 0.05. Each dot represents an enriched GO biological process with lines indicating common genes shared across GO biological process gene sets. (D) Fold-change values of mitochondrial proteins grouped according to predicted import pathways in TIMM50 patient fibroblasts relative to controls in whole cell proteomics data set. Light gray dots represent the distribution of the total cellular proteome. Statistical significance indicates import pathway substrate means relative to the total cell proteome mean. (E) Fold-change values of mitochondrial proteins grouped according to predicted import pathways in TIMM50 patient fibroblasts relative to controls in whole cell proteomics data set. Light gray dots represent the distribution of the total mitochondrial proteome with the mitochondrial mean indicated. Bottom: Statistical significance of import pathway substrate means relative to the mitochondrial mean. Top: Statistical significance represents comparison of means between import pathway substrate groups. (F and G) In vitro import of TIM22 (PiC), TIM23^MOTOR^ (OTC) and TIM23^SORT^ (SURF1 and SCO2) substrates into mitochondria isolated from HEK293 cells in the presence (black circle) or absence (gray circle) of membrane potential (ΔΨ) dissipated with 10 μM FCCP. Following incubation for indicated times, all samples were treated with proteinase K. Coomassie staining is presented as loading control. Empty arrowhead = precursor, filled arrowhead = mature. Quantifications (right) calculated as percentage of final control timepoint abundance normalized to Coomassie staining. (H) Pie charts of OXPHOS complexes according to determined import pathway. Multicolored complex II section represents SDHC and SDHD which are imported via a combined TIM23 SORT-MOTOR-OXA1L import pathway. (I and J) Correlation of OXPHOS complex median abundance with median half-life in either TIMM50 patient fibroblasts (G) or TIMM50^MUT^ HEK293 (H) cells relative to controls. Abundance data were collected from whole cell proteomics dataset. Solid line represents linear regression. Data information: C, WT HEK293 Control; M, HEK TIMM50^MUT^. In D–G, data are presented as mean ± SD. *P ≤ 0.05, **P < 0.01, ***P < 0.001, ****P < 0.0001, *N* = 3 (unpaired Student’s *t* test).

Given the phenotypes observed in both TIMM50 patient fibroblasts and HEK293 TIMM50^MUT^ cells, we asked if a unifying model focused on impaired protein import could sufficiently explain the mitochondrial dysfunction caused by loss of TIMM50 function. We hypothesized that a differential impact on TIM23 substrates, that is TIM23^MOTOR^ (targeted to the matrix) or TIM23^SORT^ (targeted to the inner membrane), could explain the observed defects in TIMM50 patient fibroblasts and TIMM50^MUT^ cells. To facilitate this interrogation, we systematically categorized all nuclear encoded mitochondrial inner membrane and matrix localized proteins (based on MitoCarta 3.0^2^) into predicted import pathways (Figure 5A). Of the initial 827 proteins, 711 were deemed TIM23 complex substrates, of which 592 were predicted to be imported via TIM23^MOTOR^ and 119 via TIM23^SORT^ (Figure 5B and Supplementary Table S4). Fifty-eight proteins were designated as TIM22 complex substrates, with another 58 either being imported via alternative pathways or having insufficient information to be properly classified (Figure 5B). Gene Ontology (GO) enrichment mapping of the protein lists generated for the three major inner membrane import pathways (TIM22, TIM23^MOTOR^ and TIM23^SORT^) affirmed the accuracy of the pipeline, with the TIM22 pathway substrate list being enriched with mitochondrial transporter proteins as expected (Figure 5C). Conversely, the TIM23^SORT^ pathway was enriched with proteins involved in OXPHOS function and cristae formation, whilst the TIM23^MOTOR^ substrates largely represented proteins involved in the mitochondrial central dogma and metabolism (Figure 5C).

The finding that the TIM23^SORT^ pathway substrate list was enriched with proteins involved in OXPHOS function and cristae formation (Figure 5C) was reminiscent of the defects observed upon loss of TIMM50 function. To interrogate this further, the substrate list generated for each inner membrane import pathway (TIM22, TIM23^MOTOR^ and TIM23^SORT^) (Figure 5A and Supplementary Table S4) was annotated against our whole cell proteomic datasets for both the TIMM50 patient fibroblasts and TIMM50^MUT^ HEK293 cells (Supplementary Table S3). Consistent with our prior observation of reduced mitochondrial protein content in TIMM50 patient and TIMM50^MUT^ cells (Figure 1H and Supplementary Figure S4D), the combined pool of TIM23 substrates was significantly reduced in abundance whilst the abundance of TIM22 substrates was unchanged (Figure 5D and Supplementary Figure S5A). Furthermore, splitting the TIM23 substrate pool into separate TIM23^MOTOR^ and TIM23^SORT^ pools revealed that in both TIMM50 patient fibroblasts and TIMM50^MUT^ cells, TIM23^SORT^ substrates were more significantly reduced in abundance than TIM23^MOTOR^ substrates (Figure 5E and Supplementary Figure S5B). Conversely, within the context of the mitochondrial proteome, substrates of the TIM22 complex were increased in relative abundance to the mitochondrial mean (Figure 5E and Supplementary Figure S5B). Whilst this could represent a compensatory response, it likely is a consequence of a global TIM23 complex substrate defect skewing the mitochondrial mean (Figure 5D). Indeed, mitochondrial outer membrane (MOM) proteins were also increased relative to the mitochondrial mean in both TIMM50 patient fibroblasts and TIMM50^MUT^ cells (Supplementary Figure S5C and D). Furthermore, TIM22 substrate abundances were unchanged in the context of the total cellular proteome (Figure 5D and Supplementary Figure S5A), suggesting that TIM22 substrates were not accumulating in the cytosol. This was confirmed using carbonate extraction, which demonstrated efficient incorporation of carrier proteins (SLC25A6 and SLC25A12) into the membrane (Supplementary Figure S5E).

The more pronounced reduction in TIM23^SORT^ substrates compared to TIM23^MOTOR^ substrates provides an explanation for the manifestation of OXPHOS and cristae defects in TIMM50 patient fibroblasts as TIM23^SORT^ substrates are enriched in these pathways (Figure 5C). To obtain biochemical support for these observations, we performed in vitro import analyses using TIM22 (PiC), TIM23^MOTOR^ (OTC) and TIM23^SORT^ (SURF1 and SCO2) substrates in WT control and TIMM50^MUT^ HEK293 cells. Consistent with the proteomics data, both TIM23^MOTOR^ and TIM23^SORT^ substrates had reduced import kinetics in TIMM50^MUT^ mitochondria, with a more severe defect apparent for the TIM23^SORT^ versus the TIM23^MOTOR^ substrates (Figure 5F). The import of the TIM23^MOTOR^ substrate OTC was reduced to 82% of control, whilst the TIM23^SORT^ substrates SURF1 and SCO2 were 67% and 62% of control, respectively (Figure 5F and G). These observations were specific to the TIM23 complex, as both the steady state levels of the TOM complex and the import kinetics of [^35^S]-hTOMM40 in the presence and absence of ATP (Supplementary Figure 6F and G) were unchanged in the TIMM50^MUT^ cells compared to WT. This suggests that loss of TIMM50 function leads to a general import defect via the TIM23 complex, which more significantly impacts substrates requiring lateral insertion into the inner membrane via TIM23^SORT^.

We queried whether increased sensitivity of TIM23^SORT^ substrates upon TIMM50 dysregulation could explain the unique OXPHOS deficiency in both TIMM50 patient fibroblasts and TIMM50^MUT^ cells. This was supported by the consistent defect we observed in the TIM23^SORT^ specific subunit ROMO1 (Figures 1I and 4C). However, assessment of the import pathways of OXPHOS complex subunits revealed that all OXPHOS complexes contain both TIM23^SORT^ and TIM23^MOTOR^ substrates (Figure 5H). Therefore, whilst the loss of ROMO1 and associated TIM23^SORT^ import provided explanations for the observed defects in complexes I, II and IV, it did not adequately explain why complexes III and V were resistant to reduced TIMM50 abundance (Figures 2A and 4D). Indeed, both complexes III and IV had a greater number of TIM23^SORT^ dependent subunits compared to TIM23^MOTOR^ (Figure 5H). Thus, we queried if the rate of protein import of substrates through the TIM23 translocase was influencing the observed deficiencies. As an indirect measure of protein import, we collated protein half-life data from Morgenstern et al.,^1^ who used a proteomic approach to determine the protein half-lives of over 800 mitochondrial proteins. Combining these half-life data with our substrate annotations of TIM23^MOTOR^, TIM23^SORT^ and TIM22, we observed no statistically significant difference in the average protein half-life of these different substrate classes (Supplementary Figure S5H), suggesting that the more severe defect in TIM23^SORT^ substrates was not because of an increased rate of import. Due to the global reliance of OXPHOS complexes on TIM23^SORT^ for subunit import (Figure 5H), we questioned if defect severity in OXPHOS complexes was related to total complex half-life, rather than the individual subunit half-life. Indeed, we found that OXPHOS complex abundance in both TIMM50 patient fibroblasts and TIMM50^MUT^ cells positively correlated with complex half-life (Figure 5I and J). These data suggest that complexes III and V do not present with a significant abundance defect in TIMM50 disease models because their increased complex half-life is able to overcome any apparent defects that would arise in steady state abundance due to reduced TIM23 complex import. Hence, the observation that complexes I, II and IV have increased turnover relative to complexes III and V and thus present with a more severe defect is supported by existing measurements of OXPHOS complex half-life.^38^

In summary, we show that mitochondrial dysfunction manifesting upon the loss of TIMM50 is determined by at least two factors: (a) pathway reliance on TIM23^SORT^ vs TIM23^MOTOR^ for protein import, and (b) the rate of protein import into mitochondria. These observations can be used to explain the defects in cristae and OXPHOS complexes due to the reliance of certain protein complexes on higher import rates via TIM23^SORT^.

## Discussion

Our data provide insight into the molecular mechanisms of mitochondrial dysfunction associated with pathogenic variants in *TIMM50*. Using an untargeted proteomic approach to explore the consequences of TIMM50 functional loss on the mitochondrial proteome in both TIMM50 patient fibroblasts and HEK293 cell models of disease, we find that substrates imported via the TIM23^SORT^ complex are most significantly impacted by reduced TIMM50 abundance. Ultimately, mitochondrial pathways with high reliance on TIM23^SORT^ for subunit import are most affected by loss of TIMM50, which includes two key mitochondrial processes involved in efficient energy generation: oxidative phosphorylation and mitochondrial cristae formation.

We validated a novel pathogenic variant in *TIMM50* identified in a pediatric patient born to consanguineous parents. The onset and nature of the clinical presentation were consistent with other reported pathogenic variants in *TIMM50* causing MGCA9, including global developmental delay, optic nerve atrophy, generalized white matter loss and infantile spasms (Supplementary Table 1), although 3-MGA was not present. The novel p.(Arg113Cys) variant triggered TIMM50 protein loss, similar to previously described TIMM50 pathogenic variants,^9^^,^^11^ which induced: (a) reductions to the steady-state levels of other core TIM23 complex subunits; (b) loss of mature TIM23 complex on BN-PAGE; and (c) reduced kinetics of import via TIM23 in vitro.

Whilst human TIMM50 has diverged from its yeast homolog, no longer possessing a key protein domain involved in precursor handover,^19^ the observed consequences on TIM23 complex assembly and function in TIMM50 patient fibroblasts confirm a crucial role for TIMM50 at the complex. Potential phosphatase activity for TIMM50 in vitro has been reported, suggesting that additional roles for human TIMM50 in regulating membrane permeability exist.^19^ Although we cannot conclusively eliminate the potential influence of such additional functions, our data propose that the manifestation of mitochondrial defects in TIMM50 patient cells is primarily driven by localized impacts at the TIM23 complex and perturbations to protein import. Whether this reduced rate of import is caused by the absence of TIMM50, or rather the loss of TIM23 complex is unclear. However, TIMM50 patient fibroblasts cultured in galactose media showed a rescue in the abundance of OXPHOS complexes.^9^ Whilst growth in galactose increases demand on OXPHOS complexes for aerobic energy production,^39^ it also triggered increased levels of TIM23 complex subunits in these TIMM50 patient fibroblasts.^9^ This suggests that despite reduced TIMM50 levels, increasing TIM23 complex subunit abundance had positive outcomes on the import of TIM23 substrates.

Using proteomics, we identified key mitochondrial processes influenced by the loss of TIMM50. In line with previous work,^9^^,^^11^ we characterized a combined defect in the abundances of complexes I, II and IV of the electron transport chain. This defect did not coincide with significant reductions in the enzymatic activity of these complexes, highlighting the additional insight provided by alternative assessment methods when investigating mitochondrial disease patient fibroblasts. In addition to disruptions in OXPHOS complex abundance, we identified changes to mitochondrial ultrastructure in TIMM50 patient cells. It has previously been suggested that mitochondria are shorter and rounded, and have reduced cristae in TIMM50 patient cells, and that TIMM50 may play a direct role in cristae formation.^11^ Our proteomic data determined that several key complexes that uphold mitochondrial cristae formation, namely complex V, MICOS and the SAM complex, were impacted in TIMM50 patient fibroblasts, providing a rationale for the observed changes in mitochondrial ultrastructure. Indeed, the structural changes observed in the TIMM50 patient cells are comparable to knockdown of the MICOS subunit Mic60, whereby mitochondria swell and accumulate mtDNA.^34^ Taken together, these data suggest that defects in TIM23 complex import, underpinned by TIMM50 mutation, drive defects in key modulators of organelle energy production and structure and this underpins the clinical features associated with *TIMM50* mutation.

Over 60% of the mitochondrial proteome is imported via the TIM23 complex.^4^^,^^5^ Whilst our data demonstrate that lack of functional TIMM50 influences global TIM23 complex function, we find specifically that import via the TIM23^SORT^ pathway is impacted to a greater extent than that of the TIM23^MOTOR^ pathway. This contrasts with models of Tim50 depletion in yeast, whereby TIM23^MOTOR^ import is more dependent on Tim50.^16^^,^^40^ The mechanism(s) underscoring this phenotype are unclear, but we postulate that TIMM50 has a more central role in TIM23^SORT^ import in human mitochondria. Indeed, the most significantly impacted peripheral TIM23 complex subunit upon loss of TIMM50 was the TIM23^SORT^ subunit ROMO1. TIMM50 may have a distinct role in the import of substrates via TIM23^SORT^ that is additive to defects caused by hypothesized loss of TIMM50 receptor function. Further exploration of this TIMM50-ROMO1 axis will aid in decoding the role of TIMM50 in the pathway governing lateral release of proteins into the inner membrane.

Increased sensitivity of TIM23^SORT^ substrates appears to be the key driver of specific mitochondrial defects in the TIMM50 patient fibroblasts. The enrichment of OXPHOS subunits and proteins involved in cristae formation within the substrate profile of TIM23^SORT^ is consistent with characterization of TIMM50 patient fibroblasts in this study and others.^9–13^ Although each OXPHOS complex contains laterally inserted substrates, we did not observe any defects in complex III. Our analysis of protein and complex half-lives suggests that this is likely due to the increased half-life of complex III subunits.^1^^,^^38^ Complex V was also largely unaffected by loss of TIMM50 by proteomics, with defects in complex V dimerization only apparent with BN-PAGE analysis. We hypothesize that this is due to the loss of several single-pass transmembrane subunits at the dimerization site, which we predict to be imported via TIM23^SORT^. Beyond these subunits, we determined that most core complex V subunits are imported via TIM23^MOTOR^ and have extended half-lives, explaining why steady state complex abundances were largely unchanged in the patient.

Models of defective protein import in *Timm23* heterozygous knockout mice demonstrate a neurological phenotype consistent with human pathogenic variants in *TIMM50*.^41^ Zebrafish timm50 knockdown models also present with severe neurological defects, cellular apoptosis in the central nervous system and cardiac dysmorphia.^19^ Furthermore, inhibition of the TIM23 complex subunit timm44 in zebrafish triggers increased apoptosis and dilated cardiomyopathy.^42^ This finding is not dissimilar to *Timm50* KO mice that present with cardiac hypertrophy, elevated reactive oxygen species (ROS) and decreased activity of OXPHOS complexes I, II and IV.^43^ Moreover, antioxidant treatment reduces cardiac hypertrophy in *Timm50* KO mice, directly implicating oxidative stress in this pathology.^43^ The driving mechanism behind ROS generation in TIMM50 disease models is unclear. Our data demonstrating reduced OXPHOS complex abundance and respiration rates upon loss of TIMM50 support a model of ROS generation centered on OXPHOS dysfunction. Indeed, supercomplex assemblies are known to sequester OXPHOS associated ROS production.^44^^,^^45^ Thus, the destabilization of super complexes upon loss of TIMM50 and emergence of increased free respiratory chain complexes despite persistence in individual activities may be indicative of abnormally regulated OXPHOS complexes generating excess ROS species. Alternatively, the absence of TIMM50 at TIM23 complexes may result in constitutively active TIM23 complex leaking ions across the inner-membrane due to the hypothesized role of TIMM50 in controlling the open and closed states of the TIM23 translocases.

In summary, we have used proteomics to create a molecular map of the proteomic consequences that result from loss of functional *TIMM50*. This molecular map can be refined to help serve as a guide in diagnosis of patients with TIMM50 variants that require further functional support. Importantly, our approach has uncovered novel insight into the fundamental biology suggesting a connection of TIMM50 in the lateral release of inner membrane proteins.

## Materials and Methods

### Clinical details, genomic sequencing, and variant detection

Samples from the proband and family members were obtained after receiving written, informed consent for diagnostic or research investigations from the responsible human ethics institutional review board (Royal Children’s Hospital HREC 34228) and research was conducted according to the Declaration of Helsinki. Detailed clinical information is available in Supplementary File 1 and Supplementary Table S1.

For the proband, blood DNA was extracted, and exome sequencing (ES) was performed at a clinically accredited laboratory (Victorian Clinical Genetics Services, Melbourne, Australia).^46^ ES was performed by massively parallel sequencing using a SureSelect QXT CREv2 exome capture kit (Agilent Technologies) on an Illumina instrument with a targeted mean coverage of 100× with a minimum of 90% of bases sequenced to at least 15×. Data were processed using CPipe,^47^ in order to generate annotated variant calls within the target region (coding exons ± 2 base pairs), via alignment to the reference genome (GRCh37). Variant prioritization by a multidisciplinary team, including clinical geneticists and medical genomics scientists, was phenotype-driven as previously described.^46^ Segregation of *TIMM50* and *FDXR* variants was performed by PCR and DNA sequencing in blood DNA extracted from the parents.

### Cell lines, cell culture, and stable cell line generation

All cell lines were cultured at 37 °C with 5% CO_2_ in Dulbecco’s Modified Eagle Medium (DMEM) (Thermo Fisher Scientific) containing 1% (vol/vol) penicillin-streptomycin (Thermo Fisher Scientific) and 5–10% (vol/vol) fetal bovine serum (In vitro Technologies). Patient fibroblasts were established from a skin biopsy. Doxycycline-inducible TIMM50^WT^ rescue cell lines in both fibroblasts and HEK293 cells were generated using the Lenti-X Tet-On Inducible Expression System (Takara Bio) as previously described.^48^ TIMM50^WT^ re-expression was induced with 50 ng/mL doxycycline for 5 days.

For the generation of CRISPR-Cas9 edited cells, HEK Flp-In™ T-REx™ 293 (Thermo Fisher Scientific) were transfected with pSpCas9(sgRNA)-2A-GFP (Addgene #48138) containing a short guide RNA targeting *TIMM50* (5′-ACGCTCGTTTTGGAGCTCAC-3′) and single-cell sorted (FACSAria^TM^ III sorter, BD Biosciences) by GFP fluorescence. Clones were screened via western blotting for TIMM50 and genomically verified by Sanger sequencing of the Cas9 target site.

### Mitochondrial isolation and treatments

Mitochondria were isolated via differential centrifugation as previously described.^49^^,^^50^ Briefly, cells were harvested in phosphate-buffered saline (PBS) (137 mM NaCl, 2.7 mM KCl, 10 mM Na_2_HPO4, 1.8 mM KH_2_PO4, pH 7.4) and isolated by centrifugation at 500 × *g*, 4 °C. Cells were homogenized with a Dounce homogenizer in isolation buffer (20 mM HEPES-KOH pH 7.6, 220 mM mannitol, 70 mM sucrose, 1 mM EDTA, 0.5 mM phenylmethylsulfonyl fluoride (PMSF), and 2 mg/mL bovine serum albumin (BSA) (BSA was omitted for downstream assessment involving MSMS)) and centrifuged at 800 × *g*, 4 °C to remove nuclear debris and intact cells. Supernatant containing mitochondria was isolated and centrifuged at 12,000 × *g*, 4 °C to isolate the mitochondrial pellet. Mitochondria were resuspended in isolation buffer (omitting PMSF and BSA), with protein concentration determined using the Pierce BCA protein assay kit (Thermo Fisher Scientific). For carbonate extraction, mitochondria were resuspended in freshly prepared 100 mM sodium carbonate and incubated on ice for 30 minutes followed by centrifugation at 100,000 × *g* and 4 °C for 30 min. Separated pellet and soluble fractions were trichloroacetic acid (TCA) precipitated with 12.5% (wt/vol) TCA and analyzed by SDS-PAGE.

### In vitro import assay

NDUFV3, GC-1, PiC, OTC, SURF1, SCO2, TOMM40, TIMM50^WT^ and TIMM50^R113C^ mRNA were synthesized using a mMESSAGE mMACHINE™ SP6 transcription kit (Ambion) and translated in rabbit reticulocyte lysate (Promega) in the presence of [^35^S]-Methionine/Cysteine (PerkinElmer). Isolated mitochondria (1 mg/mL) in import buffer (250 mM sucrose, 5 mM magnesium acetate, 80 mM potassium acetate, 10 mM sodium succinate, 1 mM DTT, 5 mM ATP, 20 mM HEPES-KOH pH 7.4) were incubated at 37 °C with radiolabeled proteins in the presence or absence of 10 μM carbonyl cyanide-p-trifluoromethoxyphenylhydrazone (FCCP). Following import, reactions were treated on ice with 50 μg/mL proteinase K for 10 min then 1 mM PMSF for 5 min to stop protease activity. For ATP depletion, both radiolabeled protein lysates and mitochondria resuspended in import buffer (without the addition of ATP) were treated with 25 U/mL of apyrase prior to import. For SDS-PAGE analysis, samples were TCA precipitated and solubilized in SDS-PAGE loading buffer before heating at 65 °C for 15 min and 95 °C for 5 min (with shaking). For BN-PAGE analysis, samples were resuspended in 1% digitonin solubilization buffer and prepared for electrophoresis as describe below in *BN-PAGE electrophoresis and in-gel activity*. Following electrophoresis, gels were transferred to PVDF membrane with radioactive signal captured with a phosphor imager screen and visualized on an Amersham Typhoon Biomolecular Imager (Cytiva).

### Radiolabeling of mitochondrial translation products

Radiolabeling of cultured HEK293 cells was conducted as previously described.^51^^,^^52^ Briefly, cells were cultured in Met/Cys-free DMEM (Thermo Fisher Scientific) containing 1% (vol/vol) penicillin-streptomycin (Thermo Fisher Scientific), 10% (vol/vol) dialyzed fetal bovine serum (In vitro Technologies), 1 mM sodium pyruvate (Thermo Fisher Scientific), 1 × GlutaMAX (Thermo Fisher Scientific), 50 μg/mL uridine (Thermo Fisher Scientific), 7 μg/mL anisomycin (Sigma-Aldrich), and 7 μCi [^35^S]-Methionine/Cysteine (PerkinElmer) for 2 h. Radiolabeling was quenched with 10 μM methionine for 15 min. Labelling media was replaced with standard culturing DMEM for 0, 3, or 24 h. Labelled cells were harvested by centrifugation at 800 × *g*, 4 °C and pellets stored at −20 °C. 50 µg of isolated mitochondria were analyzed using SDS-PAGE on 10–16% tris-tricine gels. Following electrophoresis, gels were transferred to polyvinylidene fluoride (PVDF) membrane with radioactive signal captured with a phosphor imager screen and visualized on an Amersham Typhoon Biomolecular Imager (Cytiva).

### Tricine gel electrophoresis

Tris-tricine gel electrophoresis was performed as previously described.^48^^,^^53^ Briefly, tricine gel buffered solutions (1 M Tris-Cl, 0.1% [wt/vol] Sodium dodecyl sulfate (SDS), pH 8.45) containing either 10 or 16% (vol/vol) acrylamide solution (49.5% acrylamide, 1.5% bis-acrylamide) were used to pour 10–16% gels using a gradient mixer. 16% acrylamide solutions also contained 13% (vol/vol) glycerol. Polymerized gels were overlayed with a 4% (vol/vol) acrylamide tricine gel buffered solution stacking gel. Polymerization of acrylamide containing gels was catalyzed by sequential addition of tetramethylethylenediamine (TEMED) (Sigma) and 10% ammonium persulfate (APS) (Sigma). Electrophoresis was conducted with anode (50 mM Bis-Tris, pH 7.0) and cathode buffers (0.1 M Tris, 0.1 M tricine, 0.1% [wt/vol] SDS, pH 8.45). For assessment via Tris-tricine SDS-PAGE, mitochondria were heated at 95 °C in SDS-loading dye (50 mM Tris-Cl pH 6.8, 100 mM dithiothreitol (DTT), 2% [wt/vol] SDS, 10% [vol/vol] glycerol, 0.1% [wt/vol] bromophenol blue) for 5 min.

### BN-PAGE electrophoresis and in-gel activity

For BN-PAGE, mitochondria were solubilized at 1 mg/mL in 1% (wt/vol) digitonin solubilization buffer (20 mM Tris-Cl pH 7.4, 50 mM NaCl, 0.1 mM EDTA and 10% [vol/vol] glycerol) on ice for 30 min. Following clarification by centrifugation, supernatants were combined with blue native loading dye (0.5% [wt/vol] Coomassie blue G-250 (MP Biomedicals), 50 mM α-amino n-caproic acid, 10 mM Bis-Tris pH 7.0) and loaded onto 4–16% acrylamide gradient gels as previously described.^48^ Electrophoresis was carried out at 4 °C overnight with anode buffer (50 mM Bis-Tris pH 7.0) and cathode buffer (50 mM tricine, 15 mM Bis-Tris, 0.02% [wt/vol] Coomassie blue G250). Cathode buffer was replaced with buffer lacking Coomassie blue G-250 once samples entered the separation gel. For in-gel detection of complex V enzymatic activity, BN-PAGE gels were incubated in staining solution (35 mM Tris, 270 mM glycine, 14 mM MgSO_4_, 0.2% Pb(NO_3_)_2_, 8 mM ATP pH 7.8) overnight and staining captured on a ChemiDoc MP imaging machine.

### Immunoblotting and antibodies

Following electrophoresis, gels were transferred to PVDF membrane (0.45 μM Immobilon-P; Merck) with Owl HEP-1 Semidry Electroblotting systems (Thermo Fisher Scientific). Immunoblotting was conducted with horse radish peroxidase-conjugated secondary antibodies (Sigma-Aldrich). Primary antibodies used in this study include: TIMM50 (Proteintech; 22229-1-AP), TIMM23 (BDBiosciences; 611222), TIMM17A (GeneTex; GTX108280), NDUFA9 (Ryan Laboratory, Monash University), UQCRC1 (Abcam; ab110252), COX4I1 (Cell Signaling Technology; 4850), ATP5F1A (Abcam; ab14748), ATP5ME (Abcam; ab122241), MICOS10 (Aviva Systems Biology; ARP44801_P050), MICOS60 (Proteintech; 10179-1-AP), TOMM40 (Santa Cruz; sc-365466), TOMM22 (Santa Cruz; sc-101286), SLC25A6 (Abcam; ab154007), SLC25A12 (Abcam; ab200201) and CYCS (BDBiosciences; 556432). Immunodetection was performed on a ChemiDoc MP imaging machine (BioRad) using Clarity Western ECL Substrate (BioRad). Densitometric Quantification of Western blot signal was performed using the Image Lab software (BioRad) following the manufacturer’s instructions.

### Quantitative proteomics and data presentation

Isolated mitochondria were solubilized, reduced, and alkylated in SDC buffer (1% [wt/vol] sodium deoxycholate, 100 mM Tris-Cl pH 8.1, 40 mM chloroacetamide, 10 mM tris(2-carboxyethyl)phosphine (TCEP)) by boiling at 99 °C for 5 min and sonication (Powersonic 603 Ultrasonic Cleaner, 40 KHz on high power) for 15 min at room temperature. Proteins were digested with 1:50 (wt/wt; trypsin:protein) trypsin overnight at 37 °C. Peptide solutions were clarified by centrifugation and loaded onto SDB-RPS substrate (3MTMEmporeTM) stage-tips with isopropanol containing 1% (vol/vol) trifluoroacetic acid (TFA). Stage-tips were washed with isopropanol contaning 1% TFA, then 0.2% (vol/vol) TFA, before elution with 80% (vol/vol) acetonitrile (ACN), 5% (vol/vol) ammonium hydroxide. Eluates were dried by CentriVap SpeedVac concentrator (Labconco) and reconstituted in 0.1% (vol/vol) TFA, 2% (vol/vol) ACN for analysis.

Whole cell pellets were solubilized in 5% (vol/vol) SDS, 50 mM tetraethylammonium bromide (TEAB) pH 8.5 at room temperature and clarified by centrifugation. Solubilizations were loaded onto Micro S-TRAP columns (PROFITI) according to the manufacturer’s instructions with tryptic digests performed overnight with a 1:25 trypsin to protein ratio. Eluates were dried by CentriVap SpeedVac concentrator (Labconco) and reconstituted in 0.1% (vol/vol) TFA, 2% (vol/vol) ACN for analysis.

LC MS/MS was carried out on an OrbiTrap Eclipse Mass Spectrometer (Thermo Fisher Scientific) equipped with an Acclaim Pepmap nanotrap column (Dionex-C18, 100 Å, 75 µm × 2 cm) and Acclaim Pepmap RSLC analytical column (Dionex-C18, 100 Å, 75 µm × 50 cm). Tryptic peptides were injected into the enrichment column at an isocratic flow of 5 µL/min of 2% (vol/vol) ACN containing 0.1% (vol/vol) formic acid for 5 min applied before the enrichment column was switched in line with the analytical column. The eluents were 5% (vol/vol) dimethyl sulfoxide (DMSO) in 0.1% (vol/vol) formic acid (solvent A) and 5% (vol/vol) DMSO in 100% (vol/vol) ACN and 0.1% (vol/vol) formic acid (solvent B). The flow gradient was (i) 0–6 min at 3% B; (ii) 6–7 min, 3–4% B; (iii) 7-82 min, 4–25% B; (iv) 82–86 min, 25–40% B; (v) 86–87 min, 40–80% B; (vi) 87–90 min, 80% B; (vii) 90–95 min, 80% to 3% and equilibrated at 3% B for 10 min before the next sample injection. Full MS resolutions were set to 120,000, scanning from 350–1400 m/z in the profile mode. Full MS automatic gain control (AGC) target was 250% with an IT of 50 ms. AGC target value for fragment spectra was set at 2000%; 50 windows of 13.7 Da were used with an overlap of 1 Da. Resolution was set to 30,000 and maximum IT to 55 ms. Normalized collision energy was set at 30%. All data were acquired in centroid mode using positive polarity.

Raw data were processed with Spectronaut (v 18.1.230626.50606, Sagan) against the UniProt human database (canonical + isoforms, reviewed) acquired in March 2021. Exported protein reports were imported into Perseus (v 1.6.14.0) for statistical analysis. Prior to analysis, potential contaminants were removed with a curated list. Briefly, MS2 quantities were log_2_ transformed and grouped in sample triplicate. Proteins present in less than two samples within any group were removed. Remaining proteins were annotated with the MitoCarta 3.0 data set. For isolated mitochondria and normalized mitochondrial whole cell analysis, the “subtract row cluster” feature was used for normalization according to MitoCarta 3.0 annotation. Subsequent data sets had non-mitochondrial proteins removed. For all data sets two-sample *t* tests were performed between groups using *P* value for truncation (threshold *P* value < 0.05) and presented as volcano plots using the scatter plot function. For bar chart assessment, unlogged MS2 quantities were presented as percentage of control average with statistical significance calculated by parametric unpaired multiple *t* tests in GraphPad Prism 9.3.1. For assessment of complex abundance, log_2_ fold changes of subunits were converted to percentages of control and statistical significance was determined by parametric paired multiple *t* test. Mitochondrial content was calculated from whole cell proteomic data by summing total MS2 quantities of mitochondrial proteins relative to the sum MS2 quantity of all detected proteins. Statistical significance was determined by parametric paired multiple *t* test. Topographical mapping of proteomic log_2_ fold changes was performed as previously described.^54^ Structures used were as follows: complex I (PDB: 5LDW), complex II (PDB: 1ZOY), complex III (PDB: 1BGY), complex IV (PDB: 5B1A), complex V (PDB: 7AJD).

MSMS data have been deposited to the ProteomeXchange Consortium, via the PRIDE partner repository, with the data set identifier PXD047774

### OXPHOS enzymology

Respiratory chain enzyme activities were measured in fibroblasts by spectrophotometry as described,^55^ using enriched mitochondrial fractions with (CI, CII, CIV and CS) or without (CII + CIII and CIII) hypotonic treatment. CI was measured as rotenone-sensitive NADH:ubiquinone_1_ oxidoreductase, measuring NADH oxidation at 340 nm. CII activity was measured as succinate:ubiquinone_1_ oxidoreductase, monitoring ubiquinone_1_ reduction at 280 nm. CII + CIII was assayed as succinate:cytochrome *c* reductase, measuring cytochrome *c* reduction at 550 nm, while CIII was assayed as decylbenzylquinol:cytochrome *c* reductase, measuring cytochrome *c* reduction at 550 nm. CIV was measured as cytochrome *c* oxidase, monitoring cytochrome *c* oxidation at 550 nm. The citrate synthase (CS) catalyzed production of coenzyme A (CoA.SH) from oxaloacetate was assayed by monitoring the formation of 5-thio-2 nitrobenzoate anions at 412 nm resulting from the spontaneous reaction of free sulfhydryl groups with the thiol reagent 5,5′-dithio-bis-(2-nitobenzoic acid). Enzyme activities were calculated as initial rates (CI, CII, CII + CIII and CS) or as first-order rate constants (CIII and CIV).

### Cellular respiration measurement

Cellular oxygen consumption rates (OCR) were measured in a Seahorse Bioscience XFe96 Analyzer according to the manufacturer’s protocols. Briefly, 2.5 × 10^4^ (HEK293) or 1.5 × 10^4^ (fibroblast) cells were plated per well in XFe96 culture plates (pre-treated with poly-D-Lysine) and grown overnight under standard culture conditions. Rates were measured in non-buffered DMEM media (Agilent) containing 10 mM glucose, 1 mM sodium pyruvate and 2 mM glutamine. Measurements consisted of three cycles each of 3 min mix and 3 min measure using the following inhibitors: 2 μM oligomycin; 0.5 μM FCCP; 0.5 μM rotenone and 0.3 μM antimycin A.

Following the assay, cell numbers per well were normalized using CyQuant (Thermo Fisher Scientific). Fibroblast control data are presented as average values of five independent control cell lines. Basal OCR was calculated from the average of all measurements prior to injection of oligomycin. To calculate maximal respiration, either the initial measurement following FCCP addition was used (HEK293) or an average of all measurements (fibroblasts).

### mtDNA depletion measurement

DNA was isolated from triplicate cell pellets for each cell line using a Wizard SV gDNA purification kit (Promega). Triplicate qPCR reactions were performed for each sample using TaqMan Platinum qPCR SuperMix-UDG (Thermo Fisher Scientific) with primers and probes specific for MT-ND1 (FAM/BHQ1) and CFTR (HEX/BHQ1) as described previously^56^ in a Roche LC480 real time PCR instrument. Data were analyzed using the LC480 software version 1.5.1.62 SP3 to determine the relative abundance of mtDNA by comparing the amount of the mitochondrial gene MT-ND1 with the single-copy nuclear reference gene CFTR.

### Cell imaging

Fibroblasts were seeded onto coverslips and grown overnight. HEK293 cells were seeded on to coverslips coated with 0.01% Poly-L-Lysine (Merck). For live cell imaging, cells were incubated with 50 nM MitoTracker Deep Red (Molecular Probes) and 2 μL/mL PicoGreen (Invitrogen) for 20 min at 37 °C, 5% CO_2_. Cells were rinsed in DMEM with 10% FBS (Cell Sera), 1% (vol/vol) penicillin/streptomycin prior to mounting coverslips on microscope slides. For immunofluorescence, cells were fixed with 4% paraformaldehyde (Proscitech) diluted in PBS containing 5% (wt/vol) sucrose (Sigma) for 10 min at room temperature. Following fixation, cells were washed with PBS and permeabilized with 0.1% (vol/vol) Triton-X 100 (Sigma) in PBS. Primary antibodies were diluted in PBS containing 3% (wt/vol) BSA (Sigma) and incubated for 1 h at room temperature. Secondary antibody goat anti-mouse AlexaFluor 488 or anti-rabbit AlexaFluor 568 (Invitrogen) was diluted in PBS containing 3% BSA for 1 h at room temperature. Cells were then washed with PBS and stained with Hoechst 33258 (10 μg/mL, Sigma) and mounted on slides with mounting media (0.1 M Tris-Cl pH 8.0, 90% glycerol, 0.2 M 1,4-diazabicyclo[2.2.2]octane (Sigma)). Imaging was performed using a Leica SP8 confocal microscope equipped with 488 and 552 nm 20 mW solid state lasers, a 638 nm 30 mW solid state laser and a Direct Modulation laser diode (DMOD) 405 nm 50 mW laser. Samples were mounted on a 63X HC PL APO CS2 oil lens and images were captured with the Leica LAS X SP8 software. Laser intensity and gain were optimized for each sample. Images were processed using Fiji image analysis software (https://imagej.nih.gov/ij/).

### Bioinformatics

Gene effect data and essentiality annotations of TIM23 complex subunits were collected from http://www.depmap.org (accessed: August 25, 2023. CRISPR DepMap Public 23Q2 + Score, Chronos).

Import pathway annotation datasets were manually curated from publicly available data sets. A gene list of inner membrane and matrix localized proteins as determined by MitoCarta 3.0,^2^ was appended with the indicated datasets: Uniprot, predicted transmembrane domain; Membranome, predicted transmembrane domain; AFTM, predicted number of transmembrane domains; Membranome, bitopic; MitoFates, mitochondrial targeting sequence prediction; N-terminome, presence of cleaved n-terminal sequence.^1^^,^^57–61^ All data were accessed September 1, 2023. After excluding mtDNA encoded proteins, genes were then annotated and sorted according to the parameters outlined in the pipeline. Thresholds for each group were as follows; Transmembrane Domain, yes in either database; Number of transmembrane domains, prioritized Membranome bitopic; MTS or N-Term Cleavage, MitoFates score >0.250 or presence of n-terminal cleavage. Following annotation, pathways predicted to be imported via OXA1L,^26^^,^^27^^,^^62^ which was not considered in the pipeline, were manually annotated, and re-assigned. The full annotated data set is included in Supplementary Table S4.

GO biological process enrichment analysis of significantly up and downregulated genes and import pathway gene lists were generated with NIH DAVID functional annotation tool.^63^^,^^64^ GO biological process enrichments with >4 genes and FDR q-value <0.05 were visualized with the Cytoscape plug-in EnrichmentMap.^65^

## Supplementary Material

Supplemental Material

## Data Availability

The data sets produced in this study are available in the following databases: Quantitative proteomic MSMS data: PRIDE PXD047774 (http://www.ebi.ac.uk/pride/archive/projects/ PXD047774)
