# Supplementary material for: Reduced Protein Import via TIM23 SORT Drives Disease Pathology in TIMM50-Associated Mitochondrial Disease
Source: Mol Cell Biol. 2024 Jun 3;44(6):226–44. doi: 10.1080/10985549.2024.2353652 (PMC11204040; doi:10.1080/10985549.2024.2353652)

Supplementary Figure 1

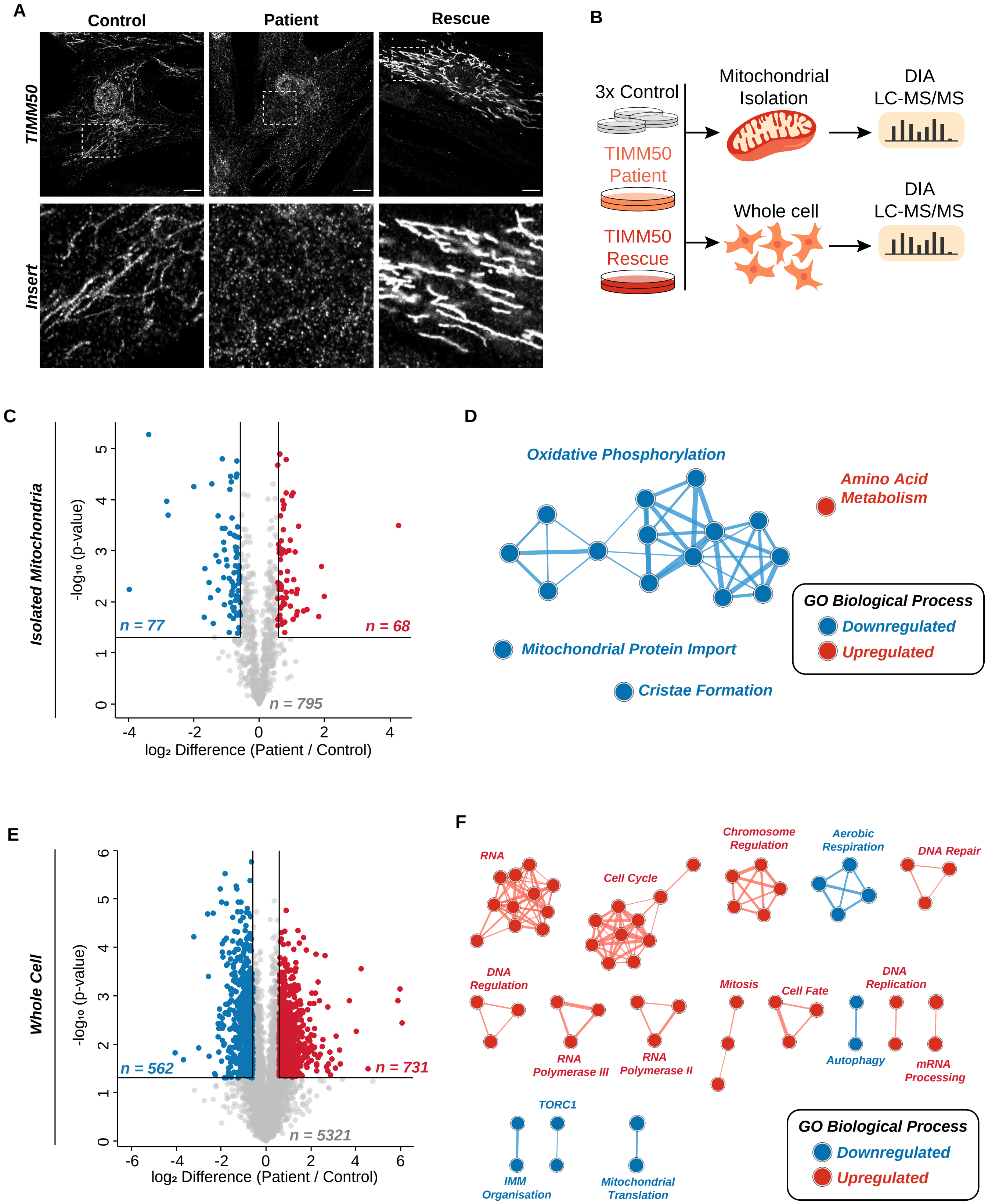

Supplementary Figure 2

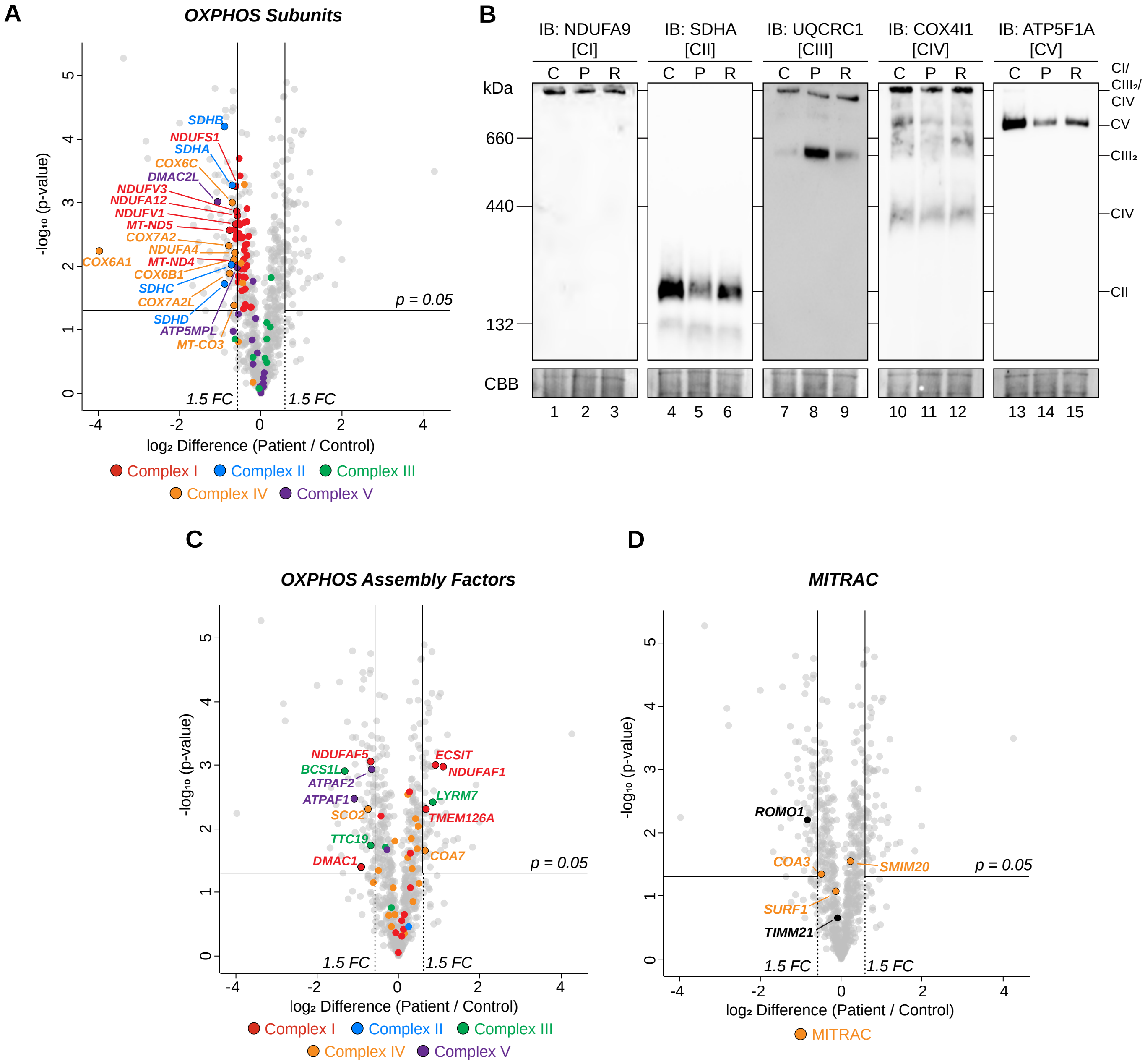

Supplementary Figure 3

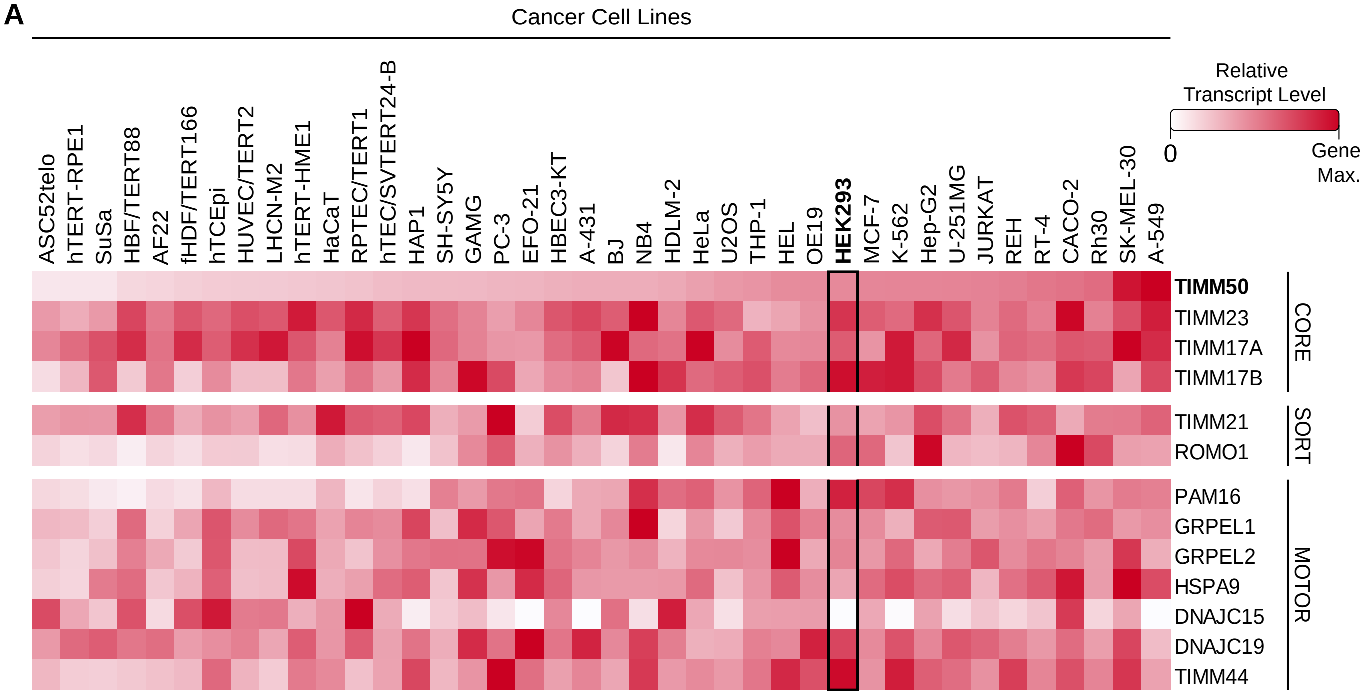

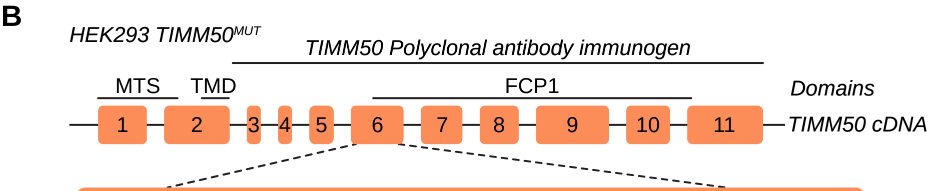

Indel	Predicted Protein Impact	Depth
c.451_460delinsCTCCCCAAGA	*p.Leu151_Leu154delinsLeuPheLysIle*	*8/10*
c.462_476del	*p.Thr155_Leu159del*	*1/10*
c.463_465del	*p.Thr155del*	*1/10*

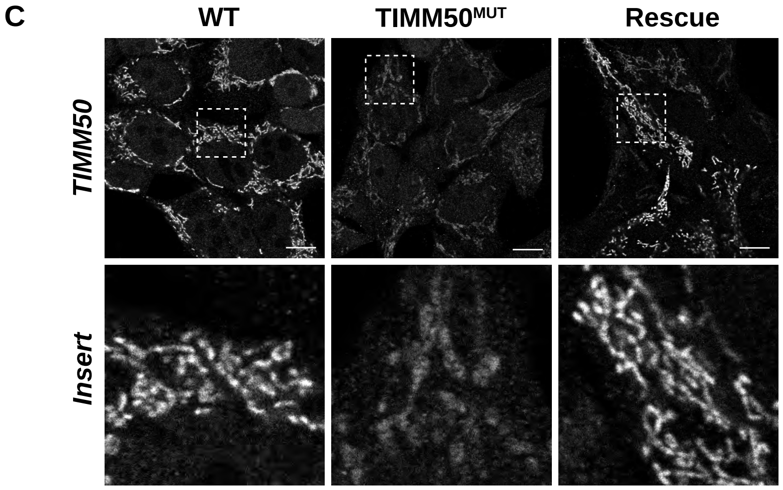

Supplementary Figure 4

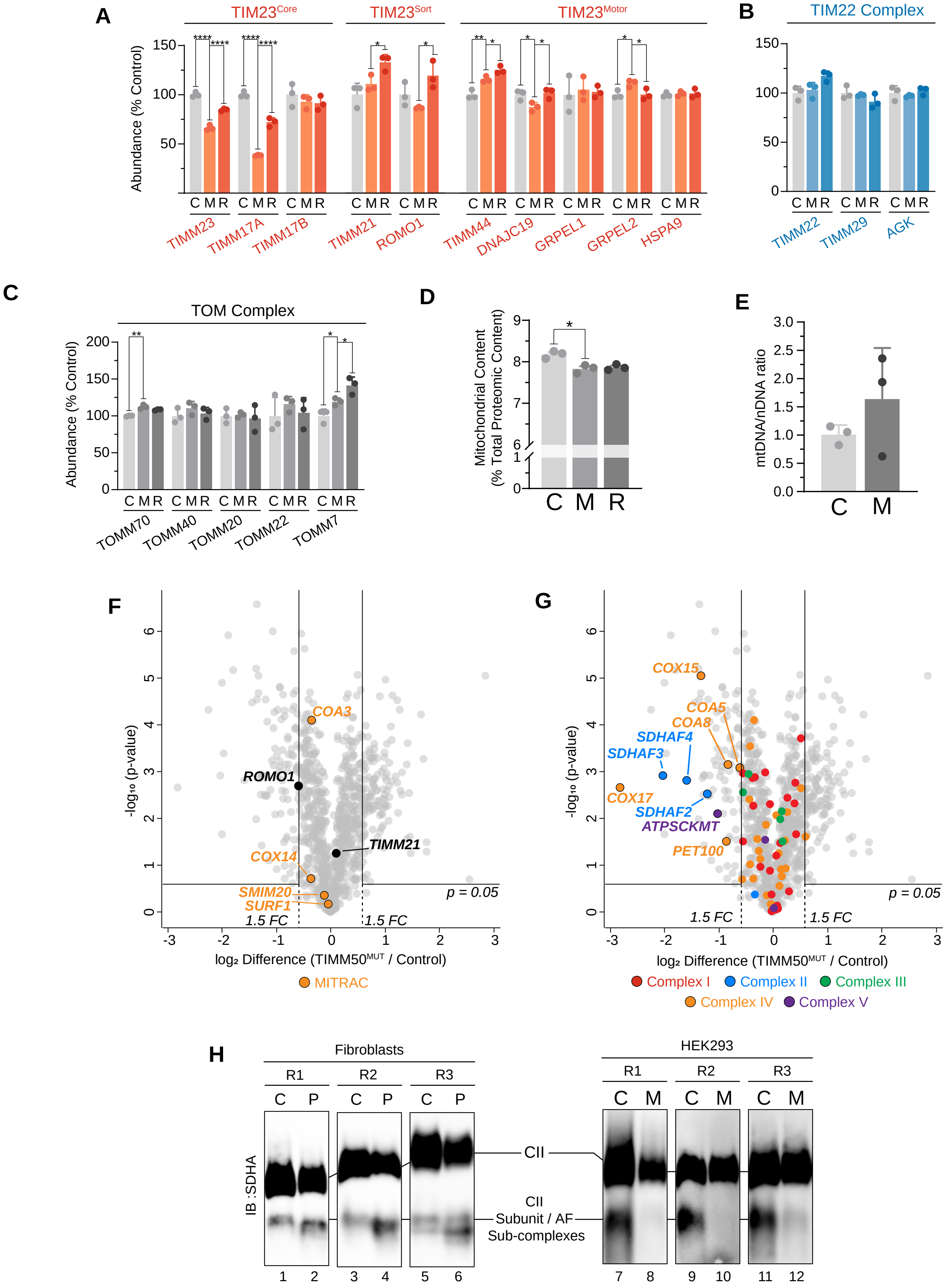

Supplementary Figure 5

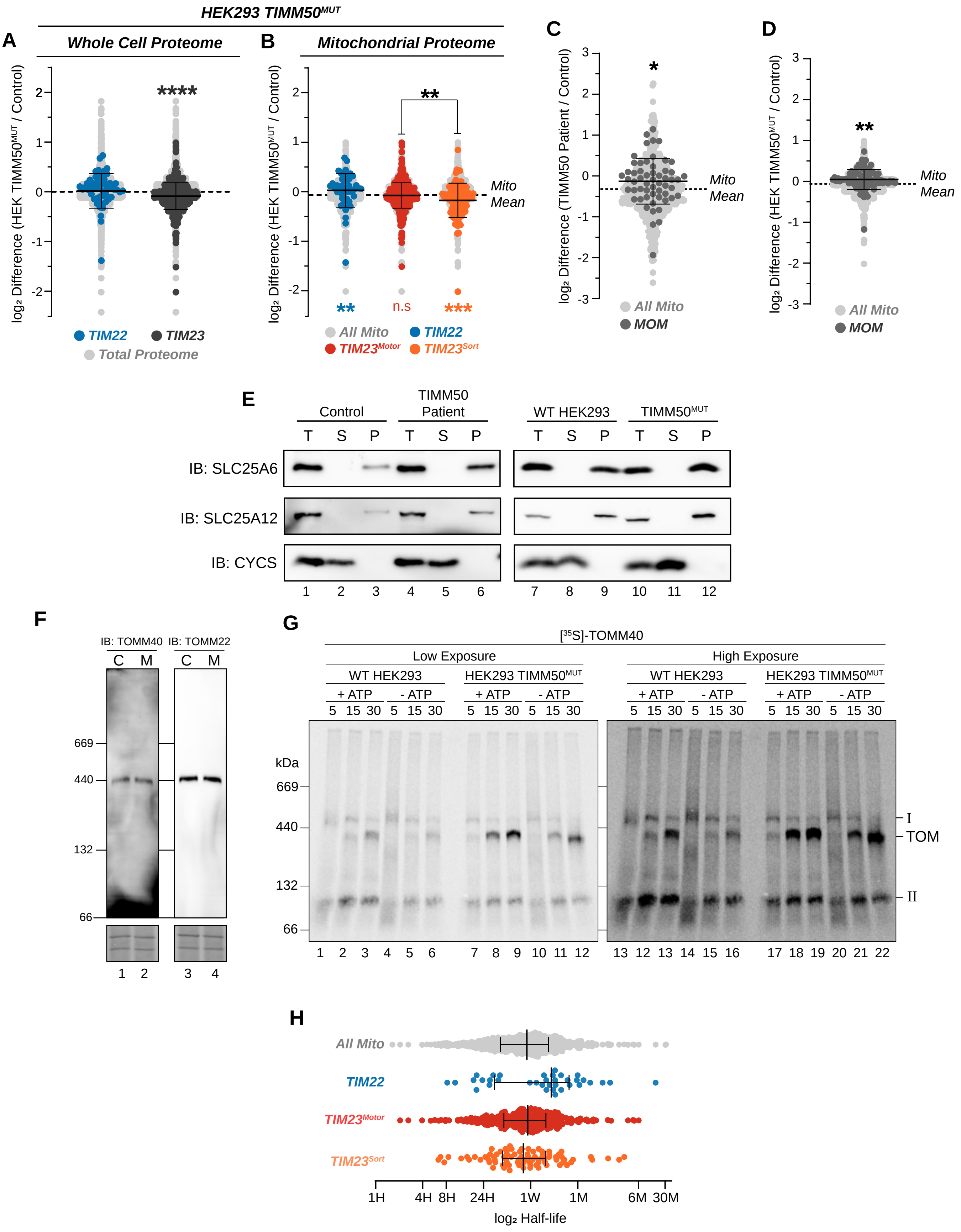

Supplement: Supplemental Material [file TMCB_A_2353652_SM0125.zip › TMCB_A_2353652_Supplementary_material/TMCB_A_2353652_Supplementary_material/suppl_data/Crameri_et_al_Supplementary_Figures_Reduced.pdf]
