# Supplementary material for: Reduced Protein Import via TIM23 SORT Drives Disease Pathology in TIMM50-Associated Mitochondrial Disease
Source: Mol Cell Biol. 2024 Jun 3;44(6):226–44. doi: 10.1080/10985549.2024.2353652 (PMC11204040; doi:10.1080/10985549.2024.2353652)
Supplement: Supplemental Material [file TMCB_A_2353652_SM0125.zip › TMCB_A_2353652_Supplementary_material/TMCB_A_2353652_Supplementary_material/suppl_data/Crameri_et_al_Supplementry_File_1.docx]

**Case Report**

This female infant is the first child of a consanguineous couple. She was delivered at 40 weeks’ gestation via emergency lower segment caesarean section for foetal distress and failure to progress. She was delivered in good condition with Apgar scores of 9 at 1 minute and 10 at 5 minutes. Birthweight was 2650 grams, head circumference 31 cm, and length 49 cm.

She presented at 4 months of age with visual inattention, global developmental delay, and cyclic upper and lower limb movements. Clinical examination revealed a rotatory nystagmus, optic nerve atrophy, generalised hyper-reflexia and upgoing plantar reflexes. A brain MRI at this stage demonstrated bilateral optic nerve atrophy and generalised white matter volume loss. Full blood count, electrolytes and liver function tests, creatine kinase, lactate, and urine metabolic screen were all normal at this stage. Of note, there was no detected excretion of 3-methylglutaconic acid in multiple urine organic acid samples. At 6 months she developed infantile spasms, with hypsarrhythmia on an EEG, and now has a refractory focal epilepsy. Currently at 5 years of age, she demonstrated profound global developmental delay functioning at the 3 months level in all developmental domains.

Whole exome sequencing identified a homozygous missense variant in exome 5 of the *TIMM50* gene (NM_001001563.5(TIMM50):c.337C>T) that was inherited from both parents. She was also heterozygous for two variants of uncertain significance in exons 6 and 12 of the mitochondrial protein *FDXR* (NM_024417.5; c.571G>A and c.1396G>A) that were both subsequently confirmed to be paternally inherited.
