# Supplementary material for: Reduced Protein Import via TIM23 SORT Drives Disease Pathology in TIMM50-Associated Mitochondrial Disease
Source: Mol Cell Biol. 2024 Jun 3;44(6):226–44. doi: 10.1080/10985549.2024.2353652 (PMC11204040; doi:10.1080/10985549.2024.2353652)
Supplement: Supplemental Material [file TMCB_A_2353652_SM0125.zip › TMCB_A_2353652_Supplementary_material/TMCB_A_2353652_Supplementary_material/suppl_data/Crameri_et_al_Supplementry_Table_1.docx]

**Supplementary Table 1. Clinical characteristics of published *TIMM50* patients**

| ***Clinical characteristics*** | ***This***  ***study*** | ***Shahrour et al,***  ***2017*** | | | | ***Reyes et al 2018*** | ***Tort et al***  ***2019*** | ***Mir et al 2020*** | ***Moudi et al***  ***2022*** | | | | | | |
| --- | --- | --- | --- | --- | --- | --- | --- | --- | --- | --- | --- | --- | --- | --- | --- |
| *Patient* | Pt | A-II-1 | A-II-3 | B-II-2 | B-II-3 | Pt | Pt | Pt | V-1 | V-2 | V-3 | V-6 | IV-15 | IV-16 |  |
| *Sex* | F | M | F | M | M | F | M | F | F | M | M | M | M | M |  |
| *Consanguinity* | + | + | | + | | - | - | + | + | | | | | | |
| *TIMM50 variants^a^* | c.337C>T;  p.(Arg113Cys) | c.446C>T;  p.(Thr149Met) | | c.340C>T; p.(Arg114Trp) | | [c.26C>A;  p.(Ser9*)],  [c.260G>C;  p.(Gly87Ala)] | [c.341G>A; p.( Arg114Gln)], [c.805 G>A; p.(Gly269Ser) | c.446C>T;  p.(Thr149Met) | c.457G>C; p.(Glu153Gln) | | | | | | |
| *Last reported age* | 5 yrs | 7 yrs | 9 yrs | 15 yrs | 10 yrs | 2 yrs (deceased) | 17 yrs | 2 yrs | 14 yrs | 18 yrs | 15 yrs | 7 yrs | 22 yrs | 20 yrs |  |
| *Seizures* | + | + | + | + | + | + | + | + | + | + | - | + | + | + |  |
| *Hypsarrhythmia or abnormal EEG* | + | + | + | + | + | + | + | + |  |  |  |  |  |  |  |
| *Abnormal brain MRI* | + | + | + | + | + | + | + | + |  | - | - | - |  |  |  |
| *Visual impairment* | + | + | + |  | - | + | + | - | - | - | - | - | - | - |  |
| *Hypotonia* |  | + |  |  | - | + |  | + |  |  |  |  |  |  |  |
| *Cardiac abnormalities* | - |  |  |  | - |  | + | + |  |  |  |  |  |  |  |
| *Developmental delay* | + | + | + | + | + | + | + | + | + | + | + | + | + | + |  |
| *Failure to thrive* | - | + | + |  | + | + | + | + | + |  |  |  |  |  |  |
| *Aggression* | - |  | + | + | + |  |  |  | + |  |  |  |  |  |  |
| *Poor/absent speech* | + | + | + | + | + |  |  |  | + | - | + | + | - | - |  |
| *Elevated lactate* | - | + | + | + | - | + | + | + | + | + | + | + | + | + |  |
| *Urine 3-MGA* | - | + | + | + | + | - | + | + |  |  |  |  |  |  |  |
| *OXPHOS complex activity* | F: CI-IV n.c. |  | M: CI-V n.c. |  | M: CI-IV n.c.; ↓ CV | M; CI, III, IV n.c.; ↓ CII | M: CI-IV n.c.; ↑ CS |  |  |  |  |  |  |  |  |

Cells left empty indicate data not provided. Abbreviations: 3-MGA, 3-methyl glutaconic aciduria; CI-V, OXPHOS complexes I-V; CS, citrate synthase; F, fibroblasts; M, muscle; n.c. no change; n.d., no data.

^a^All variants have been mapped to *TIMM50* NCBI refseq transcript NM_001001563.5
