# Supplementary material for: Reduced Protein Import via TIM23 SORT Drives Disease Pathology in TIMM50-Associated Mitochondrial Disease
Source: Mol Cell Biol. 2024 Jun 3;44(6):226–44. doi: 10.1080/10985549.2024.2353652 (PMC11204040; doi:10.1080/10985549.2024.2353652)
Supplement: Supplemental Material [file TMCB_A_2353652_SM0125.zip › TMCB_A_2353652_Supplementary_material/TMCB_A_2353652_Supplementary_material/suppl_data/Crameri_et_al_Supplementry_Table_2.docx]

**Supplementary Table 2. Enzymology of *TIMM50* patient fibroblasts**

| ***Enzyme*** | ***Activity (Range)*** | ***% Activity (Range)*** | ***% CS Ratio (Range)*** |
| --- | --- | --- | --- |
| *Complex I* | 86 (25-103) | 159 (46-191) | 125 (50-145) |
| *Complex II* | 57 (16-73) | 139 (39-178) | 110 (57-144) |
| *Complex II+III* | 67 (29-89) | 128 (56-171) | 99 (39-146) |
| *Complex III* | 24.5 (3.6-20.7) | 275 (45-233) | 212 (42-187) |
| *Complex IV* | 4.12 (0.8-8.5) | 98 (19-202) | 79 (45-170) |
| *Citrate Synthase* | 193 (64-235) | – | – |

The activities of complexes I, II, II+III and citrate synthase (CS) are expressed as nmol/min/mg, complexes III and IV are expressed as /min/mg, along with control ranges. % Activity represents data as a percentage of control mean and % CS ratio represents data as a percentage of control value relative to CS activity.
