# Supplementary material for: Reduced Protein Import via TIM23 SORT Drives Disease Pathology in TIMM50-Associated Mitochondrial Disease
Source: Mol Cell Biol. 2024 Jun 3;44(6):226–44. doi: 10.1080/10985549.2024.2353652 (PMC11204040; doi:10.1080/10985549.2024.2353652)
Supplement: Supplemental Material [file TMCB_A_2353652_SM0125.zip › TMCB_A_2353652_Supplementary_material/TMCB_A_2353652_Supplementary_material/suppl_data/Supplementary Figure Legends.docx]

# Supplementary Figure Legends

## Supplementary Figure 1

A Confocal microscopy of fibroblasts immunostained for TIMM50. Insert shows enlargement of boxed area, scale bar = 10 μm.

B Outline of proteomic pipeline for assessment of patient fibroblast cells

C Quantitative proteomic volcano plot of isolated mitochondria from TIMM50 patient fibroblasts relative to controls. Indicated are significantly changed proteins (F.C > 1.5, p-value <= 0.05) used for GO Biological Process assessment (C). Indicated n values represent number of proteins within each group (blue = downregulated, red = upregulated, grey = total)

D GO Biological Process network map of significantly upregulated (red) and downregulated (blue) proteins in TIMM50 patient fibroblast mitochondrial proteomics. Each dot represents an enriched GO Biological Process, with lines indicating common gene sets between groups. Clusters are labelled following manual curation.

E Quantitative proteomic volcano plot of whole cells from TIMM50 patient fibroblasts relative to controls. Indicated are significantly changed proteins (F.C > 1.5, p-value <= 0.05) used for GO Biological Process assessment (E). Indicated n values represent number of proteins within each group (blue = downregulated, red = upregulated, grey = total)

F GO Biological Process network map of significantly upregulated (red) and downregulated (blue) proteins in TIMM50 patient fibroblast whole cell proteomics. Each dot represents an enriched GO Biological Process, with lines indicating common gene sets between groups. Clusters are labelled following manual curation.

## Supplementary Figure 2

A Quantitative proteomic volcano plot of isolated mitochondria from TIMM50 patient fibroblasts relative to controls with OXPHOS subunits (MitoCarta 3.0) indicated. Labelled are significantly altered (F.C > 1.5, p-value <= 0.05)

B BN-PAGE of mitochondria isolated from fibroblast cells and solubilised in 1% digitonin probing for OXPHOS complexes. Coomassie staining (CBB) is presented as loading control.

C-D Quantitative proteomic volcano plot of isolated mitochondria from TIMM50 patient fibroblasts relative to controls. Shown are either (C) OXPHOS assembly factors (MitoCarta 3.0) or (D) MITRAC related proteins.

## Supplementary Figure 3

A Transcript expression of TIM23 complex subunits across common cell culture lines. Cell lines are ordered according to TIMM50 transcript expression and grouped into TIM23 complex core, sort and motor proteins. HEK293 cell data are bolded and outlined with black rectangle. Data retrieved from Human Protein Atlas (https://www.proteinatlas.org) on 20^th^ November 2023 ^68^.

B Details of indels introduced by CRISPR-Cas9 editing of *TIMM50* in HEK293 cells as determined by Sanger sequencing and predicted protein impact. Indicated in schematic are key domains of TIMM50 and immunogen used to generate polyclonal TIMM50 antibody used in this study.

C Confocal microscopy of HEK293 cells immunostained for TIMM50. Inset shows enlargement of boxed area, scale bar = 10 μm.

Data information: MTS, mitochondrial targeting sequence; TMD, transmembrane domain; FCP1, FCP1-like core domain.

## Supplementary Figure 4

A-C Translocase subunit abundance as determined by mitochondrially normalised whole cell proteomics of HEK293 cells. Values are presented as a percentage of control averages.

D Determination of mitochondrial protein content in whole cell proteomic data from HEK293 cells. Total LFQ values of mitochondrial proteins were summed and presented as a percentage of total LFQ values of all proteins.

E Quantitative PCR determination of mtDNA relative to nuclear DNA (nDNA) in HEK293 cell lines

F-G Quantitative proteomic volcano plot of isolated mitochondria from TIMM50^MUT^ HEK293 cells relative to controls. Shown are either (E) MITRAC or (F) OXPHOS assembly factors (MitoCarta 3.0)

H BN-PAGE of mitochondria isolated from HEK293 or fibroblast cells and solubilised in 1% digitonin, probing for complex II and sub-assembly complexes with antibody against SDHA.

Data information: C, Control; P, TIMM50 Patient; R, TIMM50 Patient / TIMM50^WT^ Rescue; IB, Immunoblot; R1-3, replicates 1-3. In (A-E), data are presented as mean ± SD. In (A-D) **P* ≤ 0.05, ***P* < 0.01, ****P* < 0.001, *****P* < 0.0001, n = 3 (Unpaired Student's t-test).

## Supplementary Figure 5

A Fold-change values of mitochondrial proteins grouped according to predicted import pathways in TIMM50^MUT^ HEK293 cells relative to controls in whole cell proteomics dataset. Light grey dots represent the distribution of the total cellular proteome. Statistical significance indicates import pathway substrate means relative to the total cell proteome mean.

B Fold-change values of mitochondrial proteins in whole cell proteomic dataset grouped according to predicted import pathways in TIMM50^MUT^ HEK293 cells relative to controls. Light grey dots represent the distribution of the total mitochondrial proteome with the mitochondrial mean indicated. Bottom: Statistical significance of import pathway substrate means relative to the mitochondrial mean. Top: Statistical significance represents comparison of means between import pathway substrate groups.

C-D Fold-change values of mitochondrial outer membrane (MOM) proteins in (B) TIMM50 patient fibroblasts or (C) TIMM50^MUT^ HEK293 cells relative to controls in whole cell proteomic datasets. Light grey dots represent the distribution of the total mitochondrial proteome with the mitochondrial mean indicated. Statistical significance represents MOM mean fold-changes relative to the mitochondrial mean.

E Carbonate extraction of isolated mitochondria from HEK293 cells to determine membrane integration of TIM22 substrates (SLC25A6 and SLC25A12) and a soluble control (CYCS). T = total, S = supernatant, P = pellet.

F BN-PAGE and western blot of mitochondria isolated from HEK293 cells and solubilised in 1% digitonin probing for the TOM translocase with indicated antibodies. Coomassie staining is presented as loading control.

G *In vitro* import of [^35^S]-TOMM40 into mitochondria isolated from HEK293 cells in the presence or absence of ATP, analysed by BN-PAGE. Coomassie staining is presented as loading control. I and II indicate assembly intermediates whilst TOM indicates mature assembled TOM complex.

H Half-lives of mitochondrial proteins grouped by predicted import pathways.

Data information: In (A-D), data are presented as mean ± SD. In (F), data are presented as median ± IQR. In (A-D) **P* ≤ 0.05, ***P* < 0.01, ****P* < 0.001, *****P* < 0.0001, n = 3 (Unpaired Student's t-test).

# Reference

Uhlen M, Zhang C, Lee S, Sjöstedt E, Fagerberg L, Bidkhori G, Benfeitas R, Arif M, Liu Z, Edfors F, et al. A pathology atlas of the human cancer transcriptome. Science. 2017;357:eaan2507. doi:10.1126/science.aan2507.
